# Vegetal oil-based ketogenic diet improves inflammation and fibrosis in experimental metabolic dysfunction-associated steatohepatitis

**DOI:** 10.3389/fimmu.2025.1518687

**Published:** 2025-04-01

**Authors:** Alessia Provera, Naresh Naik Ramavath, Laila Lavanya Gadipudi, Cristina Vecchio, Marina Caputo, Alessandro Antonioli, Sabrina Tini, Anteneh Nigussie Sheferaw, Simone Reano, Nicoletta Filigheddu, Marcello Manfredi, Elettra Barberis, Luca Cocolin, Ilario Ferrocino, Monica Locatelli, Massimiliano Caprio, Frank Tacke, Emanuele Albano, Flavia Prodam, Salvatore Sutti

**Affiliations:** ^1^ Department of Health Sciences and Interdisciplinary Research Centre for Autoimmune Diseases, University of Piemonte Orientale, Novara, Italy; ^2^ Unit of Endocrinology, University of Piemonte Orientale, Novara, Italy; ^3^ Department of Translational Medicine, University of Piemonte Orientale, Novara, Italy; ^4^ Department of Sciences and Technological Innovation, University of Piemonte Orientale, Alessandria, Italy; ^5^ Department of Agricultural, Forestry and Food Science, University of Torino, Grugliasco, Italy; ^6^ Department of Pharmaceutical Sciences, University of Piemonte Orientale, Novara, Italy; ^7^ Department of Human Sciences and Promotion of the Quality of Life, San Raffaele Roma Open University, Rome, Italy; ^8^ Laboratory of Cardiovascular Endocrinology, Istituto di Ricovero e Cura a Carattere Scientifico (IRCCS) San Raffaele, Rome, Italy; ^9^ Department of Hepatology & Gastroenterology, Charité - Universitätsmedizin Berlin, Berlin, Germany

**Keywords:** metabolic dysfunction-associated steatotic liver disease, non-alcoholic fatty liver disease, non-alcoholic steatohepatitis, liver fibrosis, liver inflammation, ketone bodies, gut dysbiosis

## Abstract

**Background and aims:**

Metabolic dysfunction-associated steatohepatitis (MASH) represents a growing cause of liver cirrhosis and hepatocellular carcinoma (HCC). However, effective therapy for MASH is still lacking. Despite recent studies suggest that ketosis might improve MASH evolution, the mechanisms involved have not been explored since common ketogenic diets cause severe steatohepatitis in mice. In this study, we have investigated the capacity of a new-formulated ketogenic diet (KD) containing vegetal fat in improving liver alterations associated with experimental MASH.

**Methods:**

MASH was induced in C57BL/6 mice by feeding a cholesterol-enriched Western Diet (WD) for up to 16 weeks, followed by switching animals to KD for an additional eight weeks.

**Results:**

We observed that KD administration greatly increased ketone body production and significantly reduced liver and body weights. Moreover, liver proteomic analysis and functional tests evidenced an improved glucose and lipid metabolism along with insulin resistance in KD-fed mice. These metabolic effects were associated with an amelioration in MASH-associated gut dysbiosis and with an improvement of hepatic steatosis, parenchymal injury and liver fibrosis. From the mechanistic point of view mice receiving KD showed a significant reduction in liver TREM2-positive monocyte-derived macrophages forming crown-like aggregates along with a lowering in the hepatic expression of pro-inflammatory/pro-fibrogenic markers such as CCL2, IL-12, CD11b, α1-procollagen, TGF-β1, osteopontin, and galectin-3. Consistently, *in vitro* experiments showed that β-hydroxybutyrate supplementation reduced TREM2 and galectin-3 expression by cultured Raw 264.7 macrophages.

**Conclusions:**

Altogether, these results indicate that ketogenic diet based on vegetal fat effectively improves MASH metabolic derangements and steatohepatitis, and it might represent a potential therapeutic strategy in this disease.

## Introduction

One of the consequences of the worldwide diffusion of overweight and obesity is the increasing prevalence of metabolic dysfunction-associated steatotic liver disease (MASLD) according to the new nomenclature (1). MASLD is the most frequent hepatic alteration in Western and Asian countries with a prevalence in the general population of about 25% reaching up to 70-90% among overweight and obese individuals (2). Although MASLD is more frequent in middle-aged people, its prevalence is also increasing among children in relation to the diffusion of unhealthy dietary habits (3). In about 15-20% of MASLD patients, liver steatosis can progress to metabolic dysfunction-associated steatohepatitis (MASH), a condition characterized by hepatocellular damage, intralobular inflammation, and fibrosis progressing to liver cirrhosis (4). It is estimated that within eight years, 15% of MASH patients will develop clinically evident cirrhosis (4), with a death rate ascribed to MASH-related cirrhosis of about 12-25% (1). Moreover, MASLD is increasingly recognized as an important cause of hepatocellular carcinoma (HCC) (5). Growing evidence indicates that inflammatory mechanisms consequent to the liver recruitment and activation of innate and adaptive immune cells are responsible for the onset of chronic hepatic inflammation in MASH and promote the disease evolution to fibrosis/cirrhosis as well as HCC development (6, 7).

Lifestyle changes, including healthy diet and physical activity are, so far, the most effective interventions in MASLD and significantly reduce liver steatosis and hepatic inflammation, although the effects on fibrosis are controversial (8, 9). In recent years, low carbohydrate ketogenic diets (KDs) derived from the Atkins diet have been increasingly used for weight loss and the treatment of metabolic dysfunctions (10, 11). KDs could be different in calorie amount but are all characterized by a high-fat content that, together with the low-carbohydrate content, shifts the glycolytic metabolism to a lipolytic one inducing the liver production of ketone bodies, including acetoacetate and β-hydroxybutyrate (12). Besides the capacity to sustain cell energy production, ketone bodies exert a variety of favorable effects on lipid metabolism, gene expression, and oxidative stress and have specific anti-inflammatory properties improving neurodegenerative disease, tumors, and heart failure (12). Despite small-scale clinical trials suggest that ketosis might favorably impact MASLD (13), the effectiveness of KDs in reducing systemic and hepatic inflammation has not been investigated in detail since traditional ketogenic diets enriched with animal fat cause extensive steatohepatitis when administered to mice, despite a substantial weight reduction (14). Such an effect is likely due to the combined action of low protein/choline and high cholesterol content, all factors known to promote steatohepatitis in mice (14). Consistently, Long and coworkers have recently reported that cholesterol accumulation along with IL-6 overproduction and c-jun N-terminal kinase (JNK) activation are involved in promoting steatohepatitis and liver fibrosis in mice receiving KD containing animal-derived lipids (15).

To expand the use of KDs in humans, it would be important to devise new formulations and to test in rodents whether these diets are effective in mitigating systemic and hepatic inflammation and might control fibrosis. From recent data showing that ketonemia could be reached also with diets sufficient in protein and choline (16), we devised a protein and choline-sufficient KD substituting animal fat with vegetal oil and explored its effects in a mice model of MASH.

## Materials and Methods

### Mice experimental protocol

*4 weeks-old* wild-type C57BL/6J male mice were purchased from Envigo (Bresso, Italy) and kept in pathogen-free conditions for four weeks of acclimatization before use. Steatohepatitis was induced by feeding eight-week-old male mice with a high fat/carbohydrate diet (4.6 Kcal/g) enriched with 1.25% cholesterol Western Diet (WD) for 16 weeks. Control animals received a standard chow diet. After MASH induction, mice were randomly divided into two groups, one switching to choline-sufficient KD (6.7 Kcal/g) containing 0.3% as carbohydrates, 9.2% as proteins, and 90.5% as hydrogenated coconut oil and the other continuing with the same WD diet for a further eight weeks. The safety of this new KD formulation was assessed in preliminary experiments by feeding mice for 8 weeks. All the diets were purchased from Laboratorio Dottori Piccioni (Gessate, Italy). At the end of the treatments, mice were anesthetized with sevoflurane, and after checking the anesthesia depth, the blood was collected by cardiac puncture. Afterwards, the mice were euthanized by cervical dislocation. Animal experiments were performed at the animal facility of the Dept. of Health Sciences, University of Piemonte Orientale (Novara, Italy) in compliance with EU ethical guidelines for animal experimentation. The study protocols received ethical approval by the Italian Ministry of Health (authorization N° 411/2020-PR) according to the European law requirements.

### Assessment of liver injury and metabolic status

Livers were rapidly removed, rinsed in ice-cold saline, and cut into five pieces. Aliquots were immediately frozen in liquid nitrogen and kept at −80°C until analysis. Two portions of the left lobe from each liver were fixed in 10% formalin for 24h and embedded in paraffin. Serum alanine aminotransferase (ALT) levels and liver triglycerides were determined by spectrometric kits supplied, respectively, by Gesan Production SRL (Campobello di Mazara, Italy) and Sigma Aldrich (Milano, Italy). Circulating Dipeptidyl peptidase 4 (DPP4) and Osteopontin (OPN) were evaluated on serum samples using Luminex^®^ Discovery Assays and Luminex 200™ analyzer (Bio-techne, MI, IT) according to the manufacturer’s instructions. Ketone bodies were measured once a week with Keto-Diastix^®^ (Bayer, Basel, Switzerland) according to the manufacturer’s indications on mouse urine samples collected during animal handling.

### Glucose tolerance test

The glucose tolerance test (GTT) was performed following overnight fasting and the glucose load administration (1.5g/kg) via intraperitoneal injection. Blood sampling has been performed by tail vein incision with sterile needles and the glycemia has been measured at the basal state and 10, 30, 60, 90, and 120 minutes after the glucose load through a glucometer (URight, TD-4279, Munich, Germany).

### Histology and immunohistochemistry

Formalin-fixed and paraffin-embedded (FFPE) liver sections (4-µm tick) were stained with hematoxylin/eosin using a Roche Ventana HE 600 automatic staining system (Roche Diagnostics International AG, Rotkreuz, Switzerland), while collagen deposition was detected by Picro-Sirius Red staining. Sections were scored blindly for steatosis and lobular inflammation, as described (17). The extension of Sirius Red was quantified by histo-morphometric analysis using the ImageJ software (https://imagej.nih.gov/ij/). Liver macrophages were evidenced on FFPE liver sections by immunofluorescence staining with anti-F4-80 rat monoclonal (Biolegend, USA) and anti-TREM2 rabbit monoclonal antibodies (Abcam, Cambridge, UK) in combination with secondary antibody cocktail prepared depending on the primary antibodies host species. The image acquisition was performed with Zeiss Observer 7 microscope, and background subtraction was performed with the default settings using the ZEISS software ZEN 3.1 (blue edition). Single-channel grayscale pictures were further processed in FIJI. Immunohistochemistry for galectin-3 was performed using goat polyclonal antibodies provided by R&D Systems (Minneapolis, USA) in combination with a horseradish peroxidase polymer kit (Biocare Medical, Concord, CA, USA). Microphotographs were taken using a Nikon Eclips CI microscope fitted with 20x0.5 and 40x0.75 PlanFluor lens and a DSR12 camera (Nikon Europe BV, Amsterdam, Netherlands) through the NIS-Elements F4.60.00 acquisition software.

### Flow cytometry analysis of liver leukocytes

Livers were digested by type IV collagenase (Worthington, USA), and intrahepatic leukocytes were isolated by multiple differential centrifugation steps according to Weide et al. (18). The cell preparations were then subjected to red cell lysis by BD FACS™ Lysing Solution (BD Bioscience, San Jose, CA, USA) and stained using combinations of the following monoclonal antibodies: CD45 (Clone 30-F11, Cat. 12-0451-82), CD3 eBioscience, (Thermo Fisher Scientific, Milano, Italy), CD11b (Clone M1/70, Cat. 101212), F4-80 (Clone BM8, Cat. 123113, Biolegend, San Diego, CA, USA) Thermo Fisher Scientific, Milano, Italy). Sample analysis was performed using the Attune NxT flow cytometer (Thermo Fischer Scientific, Waltham, MA, USA) and data were elaborated with FlowJo™ Software (BD Biosciences, San Jose, CA, USA).

### mRNA extraction and real-time PCR

mRNA was extracted from snap-frozen liver fragments using the TRIzol™ Reagent (Thermo Fischer Scientific, Milano, Italy). cDNA was generated from 1 µg of mRNA using the High-Capacity cDNA Reverse Transcription Kit (Applied Biosystems Italia, Monza, Italy) in a Techne TC-312 thermocycler (Techne Inc, Burlington NJ, USA). Real-Time PCR was performed in a CFX96™ Real-time PCR System (Bio-Rad, Hercules, California, USA) using TaqMan Gene Expression Master Mix and TaqMan Gene Expression probes (Thermo Fischer Scientific, Milano, Italy) reported in **Supplementary Materials**. All samples were run in duplicate, and the relative gene expression was calculated as 2^-ΔCt^ over that of the β-actin gene and expressed as a fold increase over the relative control samples.

### Microbiota analysis

Mouse stool samples have been collected from cages into sterile plastic tubes once a week for the whole experimental timeframe (T0-24) and promptly frozen and stored at -80°C before microbiota analyses. The cecum samples have been collected at T24 by killing mice and isolating each gut segment along with its content in sterile conditions. The analysis has been conducted on samples collected at the beginning (T0), before (T16), and after (T17) the switching to KD and at the end time-point (T24). DNA extraction from 1000 mg of fecal samples was carried out following the SOP 07 guidelines and procedure developed by the International Human Microbiome Standard Consortium (www.microbiome-standards.org). RNAse treatment was then performed on the extracted DNA, quantified by using the QUBIT dsHS kit, and standardized at 5 ng/μL. The V3-V4 region of the 16S rRNA was amplified using the primers 16SF (5’-TCGTCGGCAGCGTCAGATGTGTATAAGAGACAG-3’) and 16SR (5’-GTCTCGTGGGCTCGGAGATGTGTATAAGAGACAG-3’) (19), according to the Illumina 16S Metagenomic Sequencing Library Preparation instructions. Amplicons were then purified, tagged, normalized, pooled, and sequenced on the Illumina MiSeq platform (2×250 bp) according to the Illumina protocols. Raw reads were imported in QIIME2 software (https://docs.qiime2.org) for denoising by the qiime dada2 denoise-paired script (20). The amplicon-sequence variants (ASVs) obtained were then used for a taxonomic assignment against the SILVA database. ASVs table was analyzed at the lower taxonomic resolution (genus or family level).

### Metabolomic analysis

Plasma short chain fatty acids (SCFAs) were analyzed by using GCxGC-TOFMS as reported in **Supplementary Materials**.

### Proteomics analysis

Changes in the liver protein pattern were performed as described in **Supplementary Materials**.

### *In vitro* experiments

Raw264.7 cells (ATCC) were cultured (8x10^5^ cells) in 6-well plates at 37°C in complete DMEM + GlutaMAX™ (Gibco, Thermo Fisher Scientific, Milano, Italy) with 5% CO_2_. For the experiments Raw264.7 cells were treated for 48h with either butyrate 50μM (Sigma-Aldrich) or vehicle (ethanol, 0,1%) and then harvested for RNA extraction.

### Data analysis and statistical calculations

Statistical analyses were performed by SPSS statistical software 26.0 for Windows (SPSS, IBM, USA) using one-way ANOVA test with Tukey’s correction for multiple comparisons or Kruskal-Wallis test for non-parametric values. Significance was taken at the 5% level. Normality distribution was assessed by the Kolmogorov-Smirnov algorithm. Statistical analysis of proteomic data was performed using MarkerView software (Sciex, Concord, Canada) and MetaboAnalyst software (www.metaboanalyst.org). Proteins were considered up- and downregulated using fold change >1.3 or <0.769 and p-value <0.05. The significance of the difference was also analyzed by non-parametric tests using the Prism v.8 software package (GraphPad Software, San Diego, CA, USA), with statistical significance taken at p <0.05. Bioinformatics analysis of proteomic data was performed using Ingenuity Pathways Analysis (IPA) software (Qiagen, Redwood City, CA, USA). ASVs tables were then imported in R-Studio for Principal component analysis and to perform the permutational multivariate analysis of similarities (ANOSIM) by the “vegan” package in R environment. Between-group differences were assessed by the Mann–Whitney test.

## Results

### Characterization of a new formulation of the ketogenic diet for mice

**Figure 1:**
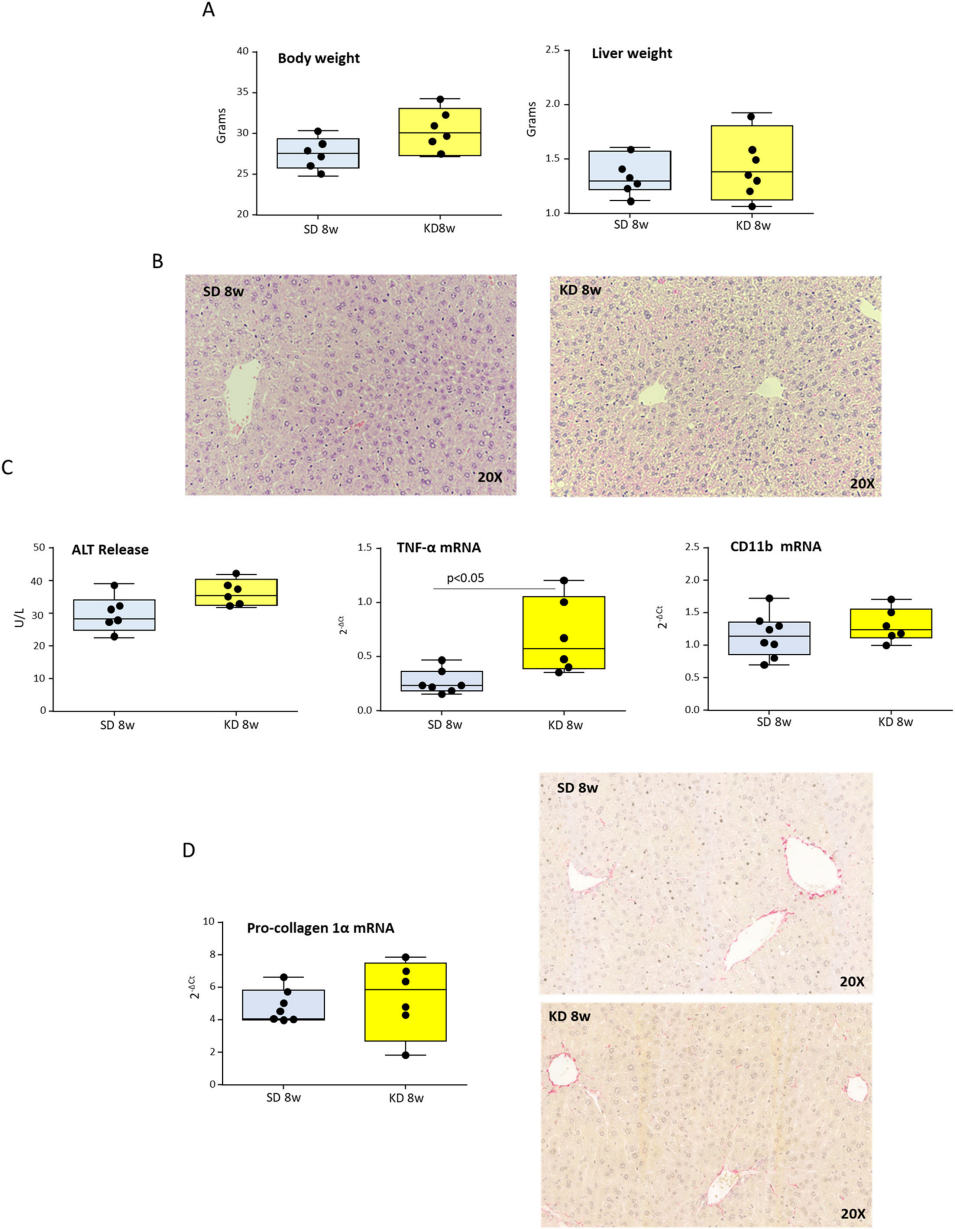
Ketogenic diet (KD) administration does not induce hepatic injury, inflammation, or fibrosis in mice. Wildtype C57BL/6 mice were fed with standard (SD) or KD diets for 8 weeks. **(A)** Body and liver weights. **(B)** Hematoxylin/Eosin staining of liver sections (magnification 20×). **(C)** Circulating levels of alanine aminotransferase (ALT) and hepatic transcripts for the inflammatory markers TNF-α and CD11b as evaluated by RT-PCR. **(D)** Intrahepatic collagen deposition as evaluated by the transcripts of procollagen-1α and the staining of liver sections with Sirius Red (magnification 20×). Dots correspond to individual animals and the boxes include the values within the 25th and 75th percentile. The horizontal bars represent the medians, while the extremities of the vertical bars (10th–90th percentile) comprise 80% of the values.

For assessing the effects of ketosis on rodent MASH evolution, we devised a choline-sufficient KD in which fat consisted of coconut oil hydrogenated to obtain a consistency suitable for shaping pellets. To avoid misleading interpretations due to caloric restriction rather than ketogenesis boosting in preliminary experiments we tested the diet palatability by comparing mice fed *ad libitum* with the standard or ketogenic diets for 8 weeks. In this time frame, we did not observe significant differences regarding food consumption (2.99 ± 0.14 vs. 2.98 ± 0.19 g/die; n.s) and body or liver weights (**Figure 1A**) while, as expected, urine ketone bodies were significantly increased in KD-fed mice as compared with those receiving standard diet (∼40 mg/ml vs. 0 mg/ml, p<0.0001). Since previous studies have shown that KD diets can cause steatohepatitis (14), histological and biochemical analyses were performed on mice receiving our new KD. Although KD-fed mice showed a slight but not statistically significant increase in serum alanine aminotransferase (ALT) (**Figure 1C**), histological analysis evidenced negligible steatosis without signs of hepatocellular necrosis and portal/lobular inflammation or fibrosis (**Figures 1B, C**). Furthermore, differently from what observed by Long and coworkers (15) our vegetal oil containing KD did not cause c-jun N-terminal kinase (JNK) activation (not shown). On the same vein, hepatic transcripts for genes commonly up-regulated in MASH showed an increase only for TNF-α, while those for the leukocyte marker integrin αM (ITGAM; CD11b) and for fibrosis-related marker pro-collagen 1A1 (COL1A1) were unaffected (**Figures 1B, C**), confirming the safety of our KD formulation in mice.

### Effects of the ketogenic diet on metabolic and pathological features of MASH

**Figure 2:**
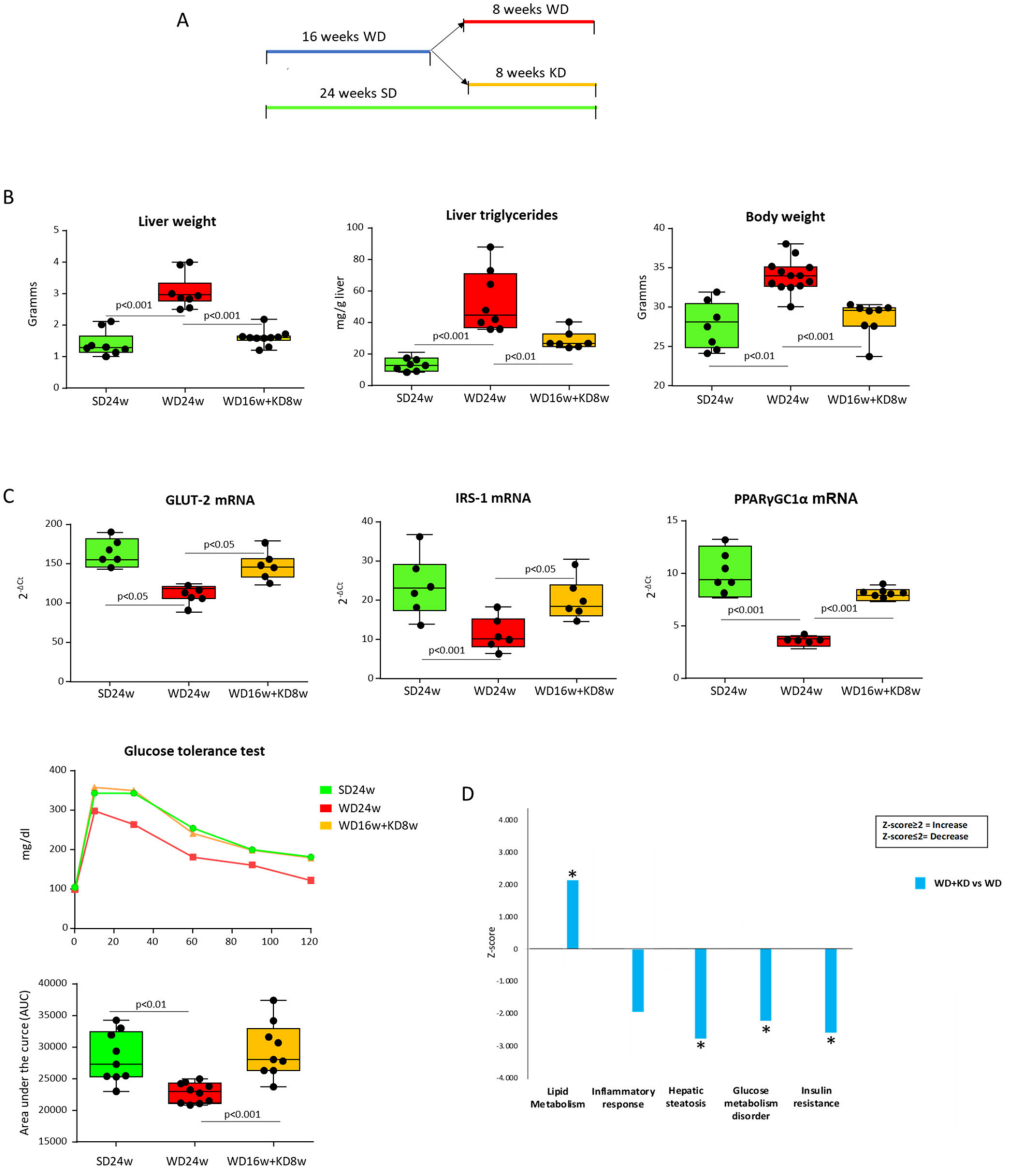
Ketogenic diet administration modifies hepatic metabolic functions in mice with MASH. MASH was induced in 8-weeks old wildtype C57BL/6 mice by 16 weeks feeding with Western diet before the switching to ketogenic diet for further 8 weeks (WD16+KD8w). Reference mice received standard diet or WD for 24 weeks (SD24w or WD24w, respectively). **(A)** Experimental Plan. **(B)** Changes in body and liver weights and hepatic triglyceride content. **(C)** Hepatic transcripts for genes of glucose transporter 2 (GLUT2) insulin receptor substrate-1 (IRS-1) and peroxisome proliferator-activated receptor-γ coactivator 1-α (PPARγC1α). RT-PCR values are expressed as fold increase of 2^-ΔCT^ after normalization to the β-actin gene. Glucose tolerance test (GTT) was assessed as area under the curve (AUC). Dots correspond to individual animals and the boxes include the values within the 25th and 75th percentile. The horizontal bars represent the medians, while the extremities of the vertical bars (10th–90th percentile) comprise 80% of the values. **(D)** Ingenuity pathway analysis (IPA) of the changes in liver proteins involved in lipid metabolism, inflammatory response, hepatic steatosis, glucose metabolism and insulin resistance in mice switching to KD as compared to those that received WD only. (*=p<0.05).

From these preliminary results, we went on to explore the effects of KD when administered to mice with established MASH. To this aim, mice were fed *ad libitum* with a cholesterol-enriched western diet (WD) for 16 weeks. As expected, WD feeding increased body and liver weights and promoted the development of extensive steatohepatitis associated with appreciable collagen deposition (**Supplementary Figure 1**). After MASH induction, mice were randomly divided into two groups, one switching to KD and the other continuing with the same WD diet for a further eight weeks (**Figure 2A**). The results were analyzed by comparing the animals switched to KD with the mice continuing the WD for 24 weeks as well as with starting conditions of mice receiving the WD for 16 weeks. We observed that switching to KD strongly reduced liver weight and lowered by 48% liver triglyceride content, while appreciably reducing body weight (**Figure 2B**). Such metabolic improvement was accompanied by the up-regulation in the hepatic transcripts of genes implicated in glucose and lipid homeostasis, such as glucose transporter 2 (GLUT2), insulin receptor substrate-1 (IRS-1), and peroxisome proliferator-activated receptor-γ coactivator 1-α (PPARγC1α) (21) (**Figure 2C**). Additionally, the switch to KD restored the physiological capacity to metabolize glucose in MASH mice, as assessed by the glucose tolerance test (GTT) (**Figure 2C**). In line with this, bioinformatic analysis comparing liver proteome profiles of mice switched to KD with those remaining on WD revealed that KD influenced different molecular pathways stimulating 77 proteins involved in lipid metabolism (Z score ≥2) while lowering (Z score ≤2), respectively, 32 proteins concerning insulin resistance, 114 proteins related to glucose metabolism, and 44 proteins associated with hepatic steatosis (**Figure 2D**, **Supplementary Table 1**).

**Figure 3:**
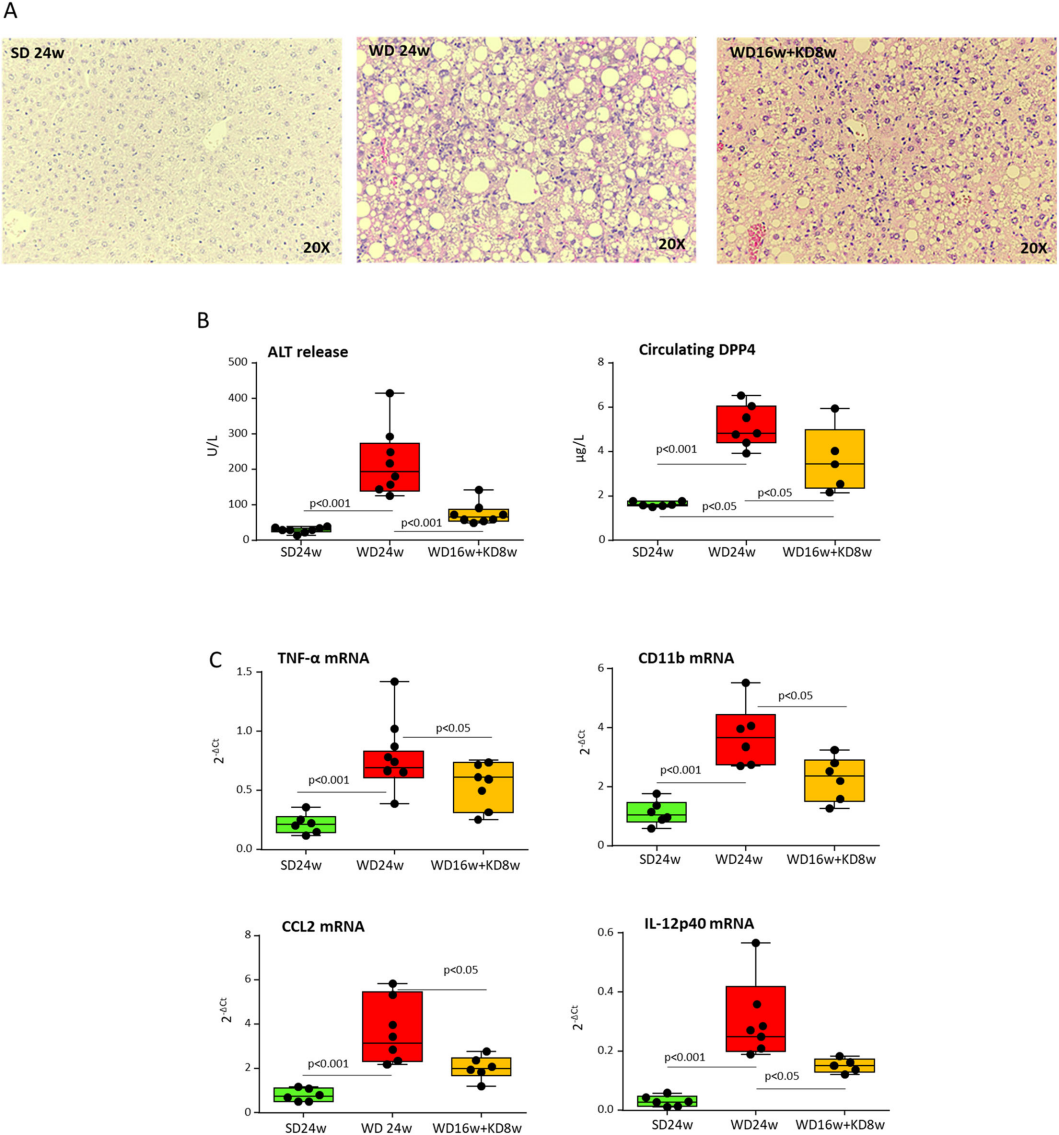
Ketogenic diet improves MASH-associated steatosis and hepatic injury in mice. MASH was induced in 8-weeks old wildtype C57BL/6 mice by 16 weeks feeding with Western diet (WD) before the switching to ketogenic diet (KD) for further 8 weeks (WD16+KD8w). Reference mice received standard diet or WD for 24 weeks (SD24w or WD24w, respectively). **(A)** Hematoxylin/eosin-staining of liver sections (magnification 20×); **(B)** Changes in circulating levels of alanine aminotransferase (ALT) and Dipeptidyl Peptidase-4 (DPP4). **(C)** Hepatic gene expression of pro-inflammatory markers TNF-α, CD11b, CCL2, and IL-12p40. RT-PCR values are expressed as fold increase of 2^-ΔCT^ after normalization to the β-actin gene. Dots correspond to individual animals and the boxes include the values within the 25th and 75th percentile. The horizontal bars represent the medians, while the extremities of the vertical bars (10th–90th percentile) comprise 80% of the values.

These changes impacted on MASH features since morphological analyses in hematoxylin/eosin-stained liver sections (**Figure 3A**) showed that KD administration significantly reduced the histological scores for hepatic steatosis (2.5 ± 0.5 vs. 0.5 ± 0.6 arbitrary units; p<0.005) and lobular inflammation (2.0 ± 0.3 vs. 0.5 ± 0.2 arbitrary units; p<0.01). Furthermore, KD significantly improved hepatic injury as assessed by ALT release as well as by circulating Dipeptidyl Peptidase-4 (DPP4) (**Figure 3B**), a metabolic marker strictly associated to MASLD/MASH in both rodents and humans (22). Consistently, the improvement of parenchymal injury was accompanied by a lowering in the gene expression of pro-inflammatory markers TNF-α, CCL2, IL-12p40 and CD11b (**Figure 3C**). The beneficial effects of KD on MASH evolution were also evident when liver damage and inflammation in mice receiving KD were compared to those of mice before diet switching (**Supplementary Figure 2**).

### Ketogenic diet modifies gut microbiota

**Figure 4:**
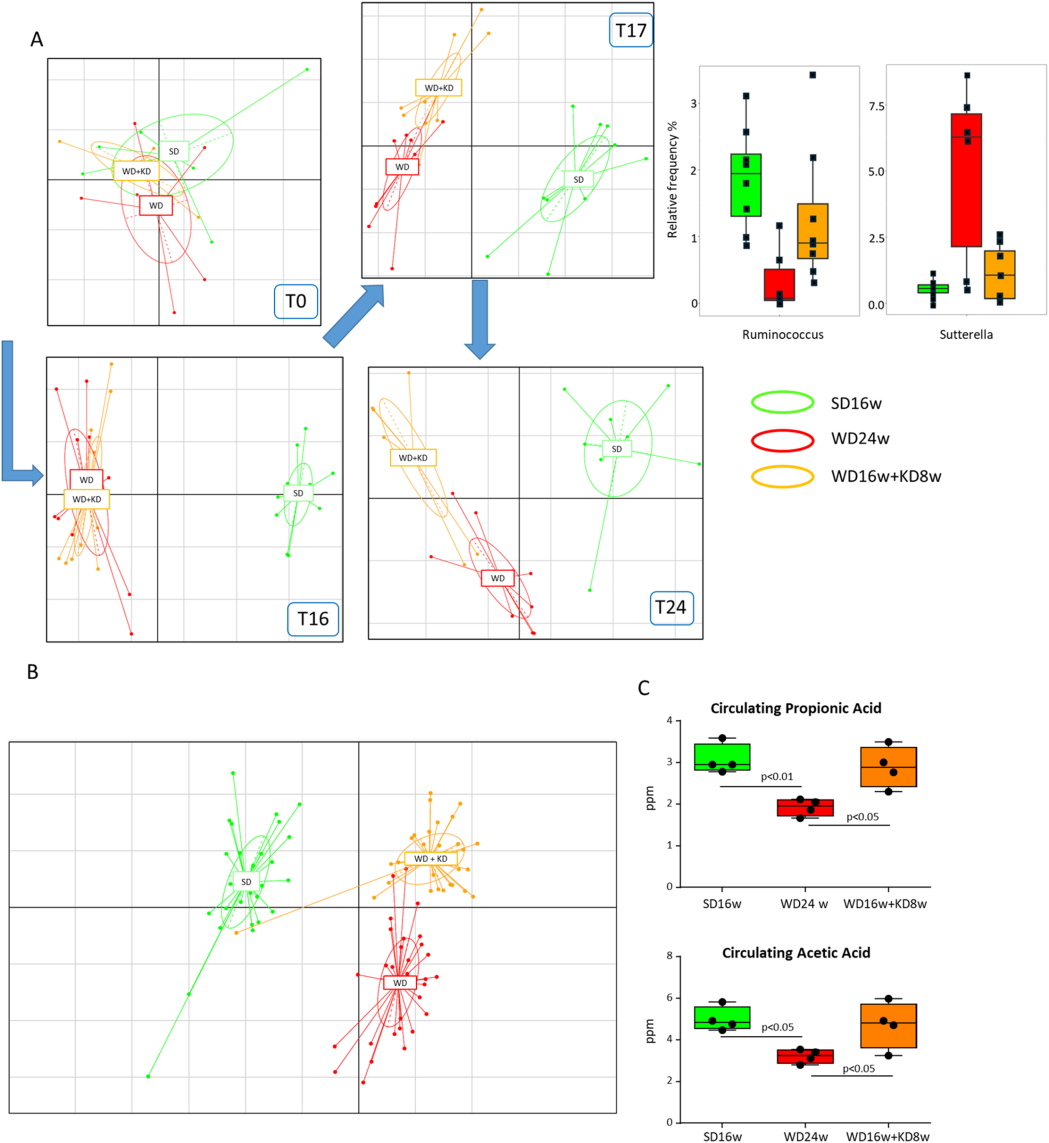
Ketogenic diet administration reshapes the gut microbiota in mice with MASH. MASH was induced in 8-weeks old wildtype C57BL/6 mice by 16 weeks feeding with Western diet before the switching to ketogenic diet (KD) for further 8 weeks (WD16+KD8w). Reference mice received standard diet or WD for 24 weeks (SD24w or WD24w, respectively). **(A)** Principal component analysis (PCA) describing the changes in stool microbiota composition at different time points during mice feeding with WD (red) as compared to those remaining in SD (green) or following the switching from WD to KD (orange). **(B)** PCA describing cecum microbiota composition in mice receiving WD, SD or after switching from WD to KD. **(C)** Changes in the serum levels of propionic and acetic acids following mice switching from WD to KD. Dots correspond to individual animals and the boxes include the values within the 25th and 75th percentile. The horizontal bars represent the medians, while the extremities of the vertical bars (10th–90th percentile) comprise 80% of the values.

MASH is often associated with alteration in the intestinal microbiota and the loss of gut barrier integrity, which can contribute to hepatic inflammation by increasing the translocation of bacterial products through the portal flow to the liver (23). From the knowledge that nutritional behaviors can influence gut microbiota composition and, in turn, hepatic inflammatory responses (24), we explored whether changes in gut microbiota could account for the anti-inflammatory properties of KD. To this aim, mouse feces were collected weekly throughout the experimental protocol and microbiota was analyzed by amplicon sequencing. According to previous studies, we observed that MASH associated with severe dysbiosis characterized by a decreased abundance of Lachnospiraceae and Ruminococcaceae and a concomitant raising of Peptostreptococcaceae, Erysipelotrichaceae, Bacteroidaceae and Sutterellaceae (25). Interestingly, the improvement of steatohepatitis in mice receiving KD led to changes in gut microbiota composition that were already appreciable after one week of treatment (**Figure 4A**). In more detail, KD increased the relative frequency of amplicon-sequence variants (ASVs) with potential anti-inflammatory properties, such as R-Ruminococcus, while decreasing ASVs displaying a potential pro-inflammatory behavior like Sutterella (**Figure 4A**) (26). The characterization of microbiota composition across the gut segments did not evidence differences in bacterial genera within the cecum, ascending, and descending colon, while they appeared more heterogeneous in the ileum regardless of the diet consumed. Therefore, we did not further consider this portion of the intestine and focused our attention on the colon. Principal component analysis (PCA) was performed on ASVs table in order to highlight shift in the stool microbiota as a function of the dietary regimen along time (**Figure 4A**). In detail, at the beginning of the experiment (T0) no clear separation between diet was observed, while after 16 weeks of dietary treatment, SD-fed mice showed a clear separation compared with WD- and WD+KD-fed mice, which clustered together (ANOSIM p<0.05). After 17 and 24 weeks of dietary treatment each mice group showed a well-clearly distinguishable bacterial signature and distinct stool microbiota composition, with mice clustering according to the dietary regimen. The separation of the microbiota as a function of the diet was then confirmed also in cecum samples (**Figure 4B**) (ANOSIM p<0.05). As a microbiota signature, KD promoted the enrichment of beneficial ASVs belonging to Oscillospiraceae, Ruminococcaceae, and Rikenellaceae. Mounting evidence suggests that microbiota plays an essential role in regulating metabolic and immune functions by producing short-chain fatty acids (SCFAs) (27). To explore the possibility that the health-beneficial properties of KD might be related to the modulation of SCFA production, we analyzed their serological concentrations before and after KD switching. In our hands, KD administration modified SCFA abundance by increasing the circulating levels of propionic and acetic acids by 1.2 and 1.4 folds, respectively (p<0.05) (**Figure 4C**). Conversely, no significant changes were appreciable for butanoic and pentanoic acids (0.06 ± 0.012 vs. 0.04 ± 0.031 ppm, 0.08 ± 0.001 vs. 0.08 ± 0.005 ppm, respectively; n.s.).

### Ketogenic diet impacts on liver macrophage responses associated with inflammation and fibrosis

From the observation that MASH improvement by our KD formulation associated with changes in gut microbiota impacting on liver inflammation we explored the mechanisms possibly involved. Proteomic analysis showed that among the hundreds of hepatic proteins affected by KD feeding the abundance of galectin-3 (Gal-3) was significantly down-modulated (log-fold change = -0.673; p=0.0028) in 3 out of 5 pathways considered. Gal-3 plays a fundamental role in driving MASH-associated liver fibrosis (28) being produced by a subset of liver infiltrating monocyte-derived macrophages (MoMFs) characterized by the co-expression of the triggering receptor expressed on myeloid cells 2 (TREM2) and CD9 as well as by the production of other pro-fibrogenic mediators such as osteopontin (OPN) (29). These TREM2^+^-MoMFs also form aggregates surrounding dead/dying hepatocytes, known as hepatic crown-like structures (hCLSs), the prevalence of which strongly correlates with the disease severity and the progression toward fibrosis (29).

**Figure 5:**
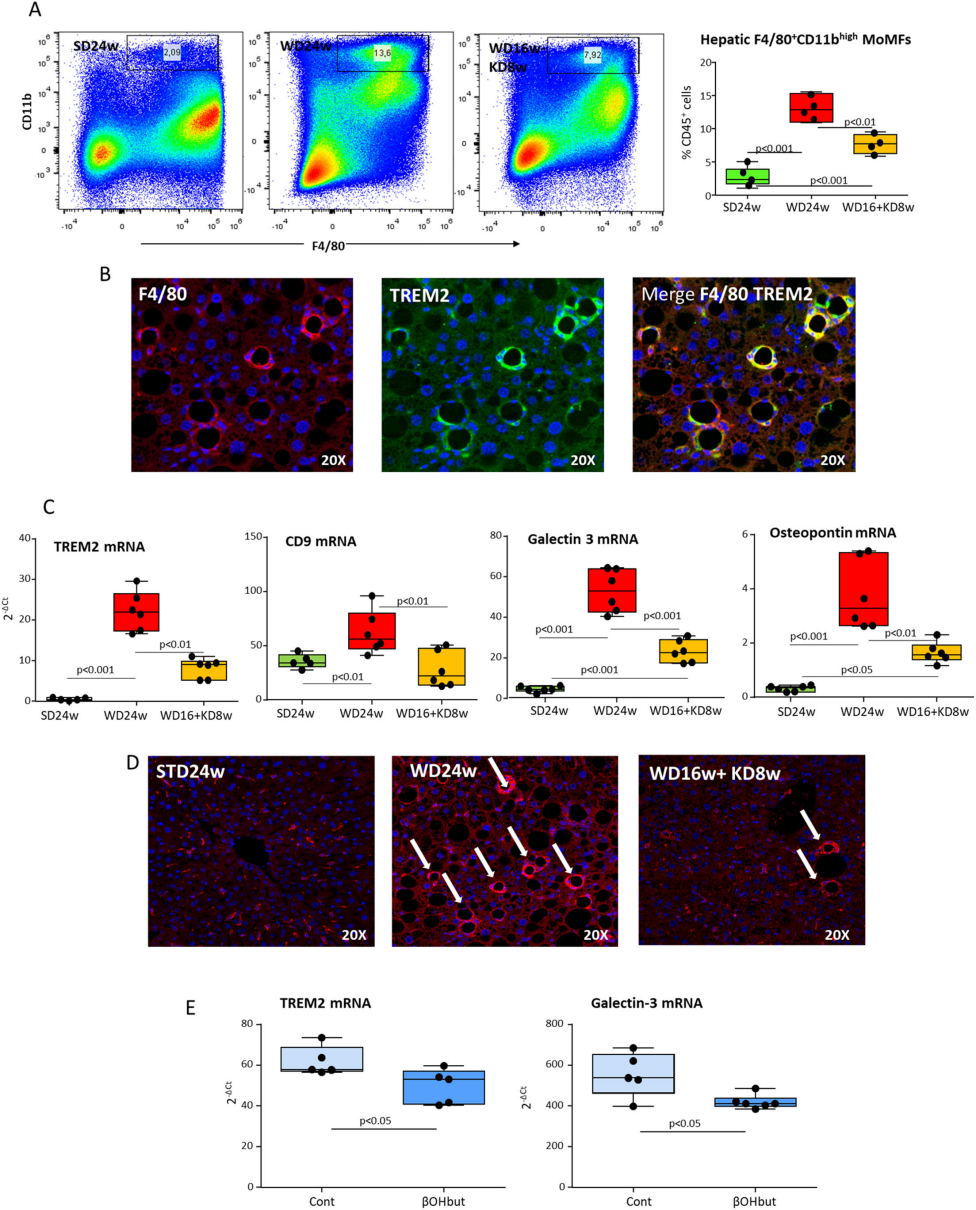
Ketogenic diet administration modulates the phenotype of hepatic macrophages and MASH-associated hepatic crown-like structures (hCLSs). MASH was induced in 8-weeks old wildtype C57BL/6 mice by 16 weeks feeding with Western diet (WD) before the switching to ketogenic diet (KD) for further 8 weeks (WD16+KD8w). Reference mice received standard diet or WD for 24 weeks (SD24w or WD24w, respectively). **(A)** Flow cytometry analysis of the distribution of CD11b^high^/F4-80^+^ monocyte-derived macrophages (MoMFs) within the liver of mice with the different dietary regiments. **(B)** Phenotypic features of liver macrophages forming hCLSs as evidenced by double immunofluorescence of FFPE liver sections stained with fluorochrome-labelled antibodies against F4-80 and TREM2. **(C)** Changes in hepatic transcripts of CD9, TREM2, galectin-3 and osteopontin. Dots correspond to individual animals and the boxes include the values within the 25th and 75th percentile, while the horizontal bars represent the medians. The extremities of the vertical bars (10th–90th percentile) comprise 80% of the values. **(D)** KD effect on the prevalence of hCLSs evidenced by immunofluorescence staining with anti-F4-80 antibodies. **(E)** Effect of β-hydroxybutyrate (β-OHbut; 50 µM) *in vitro* supplementation on TREM2 and galectin-3 expression by cultured Raw 264.7 macrophages. The experimental groups are labelled as Cont (vehicle) and *β*-OHbut and each dot represents an experimental point.

In line with these notions, multiparametric flow cytometry evidenced that MASH in WD-fed mice was characterized by an increased hepatic recruitment of pro-inflammatory CD11b^high^F4/80^int^ MoMFs (**Figure 5A**), which paralleled with the detection of F4/80^+^/TREM2^+^ hCLSs within the liver (**Figure 5B**) and the up-regulation in the transcripts for TREM2, CD9, Gal-3 and OPN (**Figure 5C**). Interestingly, the switching to KD almost halved the fraction of infiltrating CD11b^high^F4/80^int^ MoMFs (**Figure 5A**), strongly reducing the expression of TREM2^+^-cell markers (**Figure 5C**) along with the prevalence of hCLSs (23.6 ± 8.1 vs 5.5 ± 2.9 hCLSs/microscopic field; p<0.001) (**Figure 5D**). From the mechanistic point of view *in vitro* experiments using cultured Raw 264.7 macrophages showed that the supplementation with β-hydroxybutyrate (50 μM) significantly reduced TREM2 and Gal-3 expression (**Figure 5E**).

**Figure 6:**
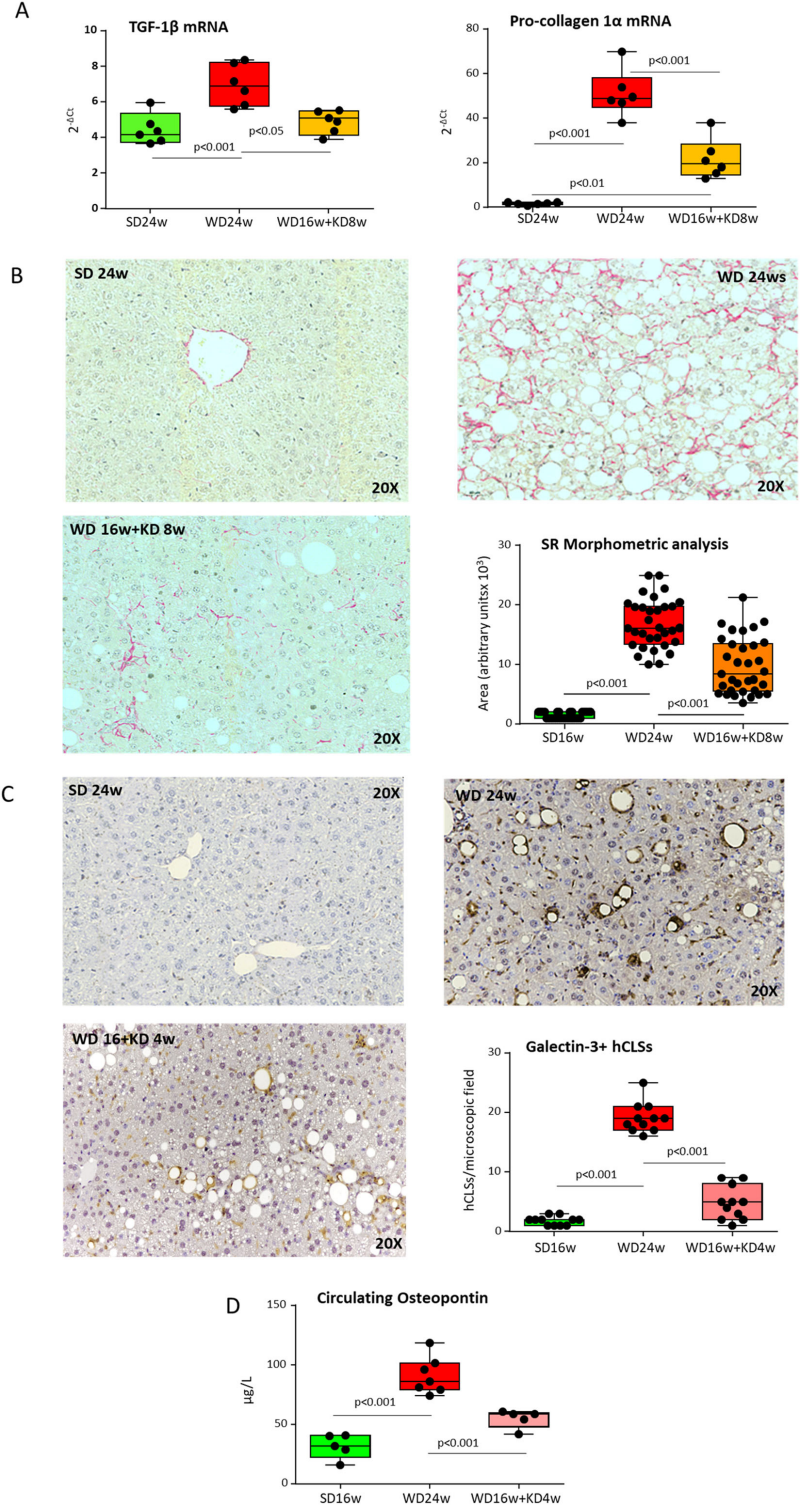
Ketogenic diet administration improves MASH-associated fibrosis in mice modulating hepatic crown-like structures (hCLSs) expressing galectin-3 and osteopontin. MASH was induced in 8-weeks old wildtype C57BL/6 mice by 16 weeks feeding with Western diet (WD) before the switching to ketogenic diet (KD) for further 4 (WD16w+KD4w) or 8 weeks (WD16+KD8w). Reference mice received standard diet or WD for 24 weeks (SD24w or WD24w, respectively). **(A)** RT-PCR analysis of the hepatic transcripts for pro-fibrogenic markers TGF-β1 and procollagen-1α. Dots correspond to individual animals and the boxes include the values within the 25th and 75th percentile. The horizontal bars represent the medians, while the extremities of the vertical bars (10th–90th percentile) comprise 80% of the values. **(B)** Effect of 8 weeks feeding with KD on liver fibrosis. The morphological analysis quantification refers to individual readings on liver sections derived from 3-4 animals for each experimental group. **(C)** Effect of 4 weeks feeding with KD on the prevalence hCLSs expressing Galectin-3 (Gal-3) as evidenced by immunohistochemistry with anti-Gal-3 antibodies and **(D)** on circulating levels of osteopontin (OPN). The morphological analysis refers to individual readings on liver sections derived from 3-4 animals for each experimental group.

The observation that KD modulated hCLSs, prompted us to investigate whether KD might also have a beneficial impact on liver fibrosis. As expected, steatohepatitis in mice fed with WD for 16 weeks raised the transcripts of the pro-fibrogenic markers procollagen 1α (Col1α) and tumor growth factor 1β (TGF-1β) (**Figure 6A**) and caused intrahepatic accumulation of collagen fibers, as quantified by Sirius Red staining of liver paraffin sections (**Figure 6B**). Noteworthy, mice receiving KD showed a significant down-modulation in the Col1α and TGF-1β gene expression along with a remarkable reduction in collagen deposition as compared to WD-fed animals, thus indicating the induction of a substantial regression of the fibrosis (**Figures 6A, B**). A marked reduction in the prevalence of Gal-3 positive hCLSs was also evident already after 4 weeks from the switching to KD (**Figure 6C**) in parallel with a lowering in circulating levels of OPN (**Figure 6D**). These effects were associated with an improvement of fibrosis and of circulating ALT and DPP4 (**Supplementary Figure 3C**), while they preceded appreciable lowering of steatosis (**Supplementary Figure 3B**) and the complete offset of hepatic inflammation (**Supplementary Figure 3D**), indicating that KD has an early and effective impact on the pro-fibrogenic mechanisms involved in MASH evolution to fibrosis/cirrhosis.

Altogether these results indicate that ketogenic diet based on vegetal fat effectively improves MASLD metabolic derangements and reshapes gut microbiota composition while improving steatohepatitis and hepatic fibrosis.

## Discussion

Despite the growing clinical relevance of MASLD, there are currently few therapeutic options. According to the European Association for the Study of the Liver (EASL) the guidelines for MASLD management rely on lifestyle changes related to dietary regimen and physical activity (30). Weight loss achieved through caloric restriction in combination with physical activity is at present the only treatment proven to ameliorate liver damage in MASLD patients without severe liver fibrosis (31). Among the dietary regimens proposed to ameliorate liver damage, ketogenic diets (KDs) are increasingly popular for treating obese patients due to their efficacy in inducing weight loss (10, 11). However, the effectiveness of KDs in MASLD/MASH has been questioned because of their high cholesterol content and the lack of essential nutrients such as choline, two factors well known to promote *per se* steatohepatitis in rodents (14). Our results addressed this question demonstrating that, by tuning nutrient composition, it is possible to devise a KD effective in improving MASH.

Hepatocytes are the main responsible for the generation of ketone bodies and, in physiological conditions, ketogenesis disposes of up to two-thirds of the lipids entering the liver. Prolonged exposure to high-fat diets, as in MASLD, progressively impairs ketogenesis likely because of oxidative stress and mitochondrial dysfunction (32). Our newly formulated KD *per se* effectively stimulated ketogenesis due to the high content of coconut oil-derived medium-chain triglycerides which are efficiently metabolized in mitochondria to produce β-hydroxybutyrate, acetoacetate and acetone (33). On the other hand, the use of vegetal oil as the main calorie source avoids cholesterol overload associated with the use of animal fat.

Ketone bodies, besides serving as energy fuel for different tissues, have been shown to exert a variety of biological functions, including the capacity to act as epigenetic modifiers of histones, thus regulating chromatin architecture and gene transcription (12). In line with ketone bodies capacity of regulating gene expression, we have observed that MASH mice switching to KD modulates multiple metabolic pathways involved in hepatic lipid and glucose metabolism as well as insulin responses. In this regard, switching to KD improves glucose tolerance, increasing GLUT2 and IRS-1 expression. This is obtained despite a modest weight decrease, suggesting that ketone synthesis can remove the excess of acetyl-CoA associated with fatty liver and hyperglycemia (34). Indeed, restoring ketone body production improves overall glycemic control even before achieving weight loss (35). Intriguingly, we have also observed that KD promotes the restoration of normal circulating DPP4, a key enzyme in glucose metabolism (36). This strengthens recent findings suggesting DPP4 as a marker of MASLD and the action of DPP4 inhibitors in hepatic fat reduction (37).

Besides improving fatty liver, we also found that, differently from simple dietary restriction, KD effectively counteracts steatohepatitis by lowering hepatic damage and lobular inflammation. These effects can be ascribed to the anti-inflammatory properties of β-hydroxybutyrate that inhibit NF-kB translocation through IκB-α degradation and triggers G-protein-coupled receptor (GPR)109A, abundantly expressed in many immune cells, including monocytes and macrophages (12). At present, hepatic macrophages are considered the main player in MASH progression to fibrosis/cirrhosis by producing pro-inflammatory mediators perpetuating hepatocyte injury and liver inflammation and by directing extracellular matrix production (6). Recent single-cell RNA sequencing analysis of liver infiltrating macrophages has evidenced the phenotype heterogeneity of these cells in both human and rodent MASH (29). The studies also outline the importance of the so-called TREM2 expressing monocyte-derived macrophages (TREM2^+^-MoMFs) (29), which localize within regions characterized by inflammation, cell death, and extracellular matrix remodeling (38). Furthermore, these cells form hepatic crown-like structure (hCLS) surrounding dying hepatocytes (29). In our hands, KD-fed mice show a significant lowering in the fraction of liver infiltrating CD11b^high^F4/80^int^ macrophages displaying TREM2^+^-MoMF features, which parallels with a reduction in hCLSs. Consistently, cultured macrophage supplementation with β-hydroxybutyrate down-modulates the expression of TREM2 and galectin-3, suggesting a specific action of ketone bodies on MASH-associated MoMFs. These findings contrast with previous data showing that KD supplementation fosters hepatic inflammation by promoting macrophage accumulation (39) and increasing the secretion of pro-inflammatory cytokines (15). However, such a discrepancy can be explained by considering that KDs employed in the studies above were rich in cholesterol, which is known to promote hepatic inflammation by stimulating macrophage activation and hCLS formation (40).

Growing evidence suggests that gut dysbiosis represents a common feature in MASLD, supporting disease progression by sustaining hepatic inflammatory responses (41). We have observed that KD reshapes MASH-associated gut microbiota promoting the enrichment of bacterial strains having anti-inflammatory properties such as *Oscillospiraceae*, *Ruminococcaceae*, and *Rikenellaceae* (42). Interestingly, the relative abundance of those bacterial families is decreased in gut microbiota signatures associated with human MASLD, thus supporting their role in maintaining liver health (41). Gut microbiota contributes to the degradation of nutrients, producing bioactive compounds that can modulate host metabolism. Among microbial-derived metabolites SCFAs such as acetate, propionate, and butyrate regulate the immune system and modulate inflammation by influencing activation/differentiation and migration of immune cells, including macrophages (23). Furthermore, nutritional behaviors influence SCFA production along with their metabolic effects. In our hands, KD not only reshapes gut microbiota composition but also significantly modulates SCFA production by raising the serological levels of propionate and acetate. Noteworthy, both these metabolites exhibit anti-inflammatory properties by influencing macrophage functional capacities (43, 44). For instance, acetate inhibits the TLR4 signaling pathway in macrophages through the up-regulation of the tripartite motif-containing protein 40 (TRIM40), reducing their responsiveness to pro-inflammatory stimuli such as damage/pathogen-associated molecular patterns (43). Overall, our results suggest that by modulating gut microbiota, KD can effectively interfere with pro-inflammatory mechanisms involved in promoting steatohepatitis even in the absence of appreciable body weight reduction.

A novel finding concerning the beneficial actions of KD in MASH concerns the improvement of hepatic fibrosis. We have observed that KD supplementation not only ameliorates steatohepatitis, the main factor responsible for stimulating hepatic collagen deposition, but also promotes the regression of already established fibrosis, as evidenced by the early reduction of overall intrahepatic collagen content. Such antifibrotic actions of KD likely involve an effect on TREM-2^+^-MoMFs, in relation to their capability of producing pro-fibrogenic mediators like OPN and Gal-3 (24). Indeed, both OPN and Gal-3 liver expression are lowered in mice receiving KD in parallel with those of fibrosis markers. The action of KD on the mechanisms supporting hepatic fibrogenesis is consistent with the data by Moore and colleagues (45), showing that dietary supplementation with the ketone ester R, S-1,3-butanediol diacetoacetate (BD-AcAc2) attenuates hepatic stellate cell (HSC) activation and hepatic fibrosis in the context of high-fat diet (HFD)-induced obesity. Conversely, our findings differ from those by Liao et al., who evidence that KD exacerbates carbon tetrachloride (CCl_4_)- or thioacetamide (TAA)-induced liver fibrosis (46). Such a discrepancy might represent a specific effect of KD in MASH-related fibrosis and might also depend on the use in Liao’s experiments of a cholesterol-rich KD (47), which is known to support hepatic fibrosis along with inflammation (48). Although the beneficial effects of ketosis on hepatic fibrogenesis have been proposed some years ago by Puchalska et al. (12) demonstrating its role in preventing tissue scarring, the present study adds new insights regarding the anti-fibrotic action of ketosis, revealing its capability to also mediate fibrosis regression. KD action on hepatic fibrosis is highly relevant because fibrotic livers are fertile ground for HCC development (5), and the severity of fibrosis is the strongest predictor for disease-specific mortality in MASLD/MASH patients (4). It is now accepted that clinical and experimental liver fibrosis can regress when the causative agent is removed. Such an effect relies on the elimination of myofibroblasts derived from activated HSCs and the progressive degradation of collagen in the fibrous scar (48). The mechanisms leading to fibrosis regression by KD have not been investigated in detail. Puchalska and colleagues (49) have reported that hepatocyte-generated acetoacetate, but not β-hydroxybutyrate, specifically down-modulates fibrogenic responses in liver macrophages *via* mitochondrial metabolism. Nonetheless, we cannot exclude that ketosis might favor macrophage phenotypic conversion to a restorative Ly6C^low^ subset, expressing phagocytosis-associated receptors and secreting matrix metalloproteases which are involved in liver matrix degradation (48). Nonetheless, it is also possible that the changes in the tissue microenvironment due to ketone bodies and microbiota-derived metabolites might modulate innate immune cell memory, which plays an important role in sustaining chronic inflammation and fibrosis (50, 51). In this latter respect, it is plausible that by counteracting the detrimental effects of the maladaptive training of myeloid cells (52), KD-induced modulation of innate immune memory could also positively impact on inflammatory comorbidities often associated with MASH. Further analyses are needed to address these unresolved issues and to gain a more detailed understanding of the mechanisms by which KD administration leads to fibrosis regression.

In conclusion, our findings suggest that the replacement of animal fat with vegetal oil in KD is not harmful in the short term and significantly ameliorates steatohepatitis by restoring essential metabolic functions, counteracting dysbiosis, and reducing the hepatic recruitment of TREM2^+^-MoMFs. Strikingly, the newly formulated KD also favors the regression of liver fibrosis. Therefore, such a dietary regimen could represent a novel potential therapeutic tool to be exploited for the treatment of MASH-associated fibrosis. Nonetheless, our study suffers from the limitation that the animal model used does not allow to investigate the long-term effects of KD. This is a relevant issue considering that dietary components increased in KDs, particularly cholesterol and saturated fats, are linked to an enhanced risk of chronic kidney diseases, cardiovascular diseases, cancer, diabetes, and Alzheimer’s disease (53). Therefore, it would be relevant to verify whether our new KD formulation could help in minimizing these potential risks.

## Data Availability

The datasets presented in this study can be found in online repositories. The names of the repository/repositories and accession number(s) can be found below: PRJNA1175602.

## References

[1] Rinella ME Lazarus JV Ratziu V Francque SM Sanyal AJ Kanwal F. A multisociety Delphi consensus statement on new fatty liver disease nomenclature. J Hepatol. (2023) 79:1542–56. doi: 10.1016/j.jhep.2023.06.003 37364790

[2] Younossi ZM Golabi P Paik JM Henry A Van Dongen C Henry L. The global epidemiology of nonalcoholic fatty liver disease (NAFLD) and nonalcoholic steatohepatitis (NASH): a systematic review. Hepatology. (2023) 77:1335–47. doi: 10.1097/HEP.0000000000000004 PMC10026948 36626630

[3] Nobili V Svegliati-Baroni G Alisi A Miele L Valenti L Vajro P. A360-degree overview of pediatric NAFLD: recent insights. J Hepatol. (2013) 58:1218–29. doi: 10.1016/j.jhep.2012.12.003 23238106

[4] Powell EE Wong VW Rinella M. Non-alcoholic fatty liver disease. Lancet. (2021) 397:2212–24. doi: 10.1016/S0140-6736(20)32511-3 33894145

[5] Huang DQ El-Serag HB Loomba R. Global epidemiology of NAFLD-related HCC: trends, predictions, risk factors and prevention. Nat Rev Gastroenterol Hepatol. (2021) 18:223–38. doi: 10.1038/s41575-020-00381-6 PMC8016738 33349658

[6] Subramanian P Chavakis T. The complex function of macrophages and their subpopulations in metabolic injury associated fatty liver disease. J Physiol. (2023) 601:1159–71. doi: 10.1113/JP283820 36825510

[7] Ramadori P Kam S Heikenwalder M. T cells: Friends and foes in NASH pathogenesis and hepatocarcinogenesis. Hepatology. (2022) 75:1038–49. doi: 10.1002/hep.32336 35023202

[8] Promrat K Kleiner DE Niemeier HM Jackvony E Kearns M Wands JR. Randomized controlled trial testing the effects of weight loss on nonalcoholic steatohepatitis. Hepatol (Baltimore Md). (2010) 51:121–9. doi: 10.1002/hep.23276 PMC2799538 19827166

[9] Romero-Gómez M Zelber-Sagi S Trenell M. Treatment of NAFLD with diet, physical activity and exercise. J Hepatol. (2017) 67:829–46. doi: 10.1016/j.jhep.2017.05.016 28545937

[10] Caprio M Infante M Moriconi E Armani A Fabbri A Mantovani G. Very-low-calorie ketogenic diet (VLCKD) in the management of metabolic diseases: systematic review and consensus statement from the Italian Society of Endocrinology (SIE). J Endocrinol Invest. (2019) 42:1365–86. doi: 10.1007/s40618-019-01061-2 31111407

[11] Muscogiuri G El Ghoch M Colao A Hassapidou M Yumuk V Busetto L. European guidelines for obesity management in adults with a very low-calorie ketogenic diet: A systematic review and meta-analysis. Obes Facts. (2021) 14:222–45. doi: 10.1159/000515381 PMC8138199 33882506

[12] Puchalska P Crawford PA. Multi-dimensional roles of ketone bodies in fuel metabolism, signaling, and therapeutics. Cell Metab. (2017) 25:262–84. doi: 10.1016/j.cmet.2016.12.022 PMC5313038 28178565

[13] Watanabe M Tozzi R Risi R Tuccinardi D Mariani S Basciani S. Beneficial effects of the ketogenic diet on nonalcoholic fatty liver disease: A comprehensive review of the literature. Obes Rev. (2020) 21:e13024. doi: 10.1111/obr.13024 32207237 PMC7379247

[14] Garbow JR Doherty JM Schugar RC Travers S Weber ML Wentz AE. Hepatic steatosis, inflammation, and ER stress in mice maintained long term on a very low-carbohydrate ketogenic diet. Am J Physiol Gastrointest Liver Physiol. (2011) 300:G956–67. doi: 10.1152/ajpgi.00539.2010 PMC3119109 21454445

[15] Long F Bhatti MR Kellenberger A Sun W Modica S Höring M. Low-carbohydrate diet induces hepatic insulin resistance and metabolic associated fatty liver disease in mice. Mol Metab. (2023) 69:101675. doi: 10.1016/j.molmet.2023.101675 36682412 PMC9900440

[16] Ang QY Alexander M Newman JC Tian Y Cai J Upadhyay V. Ketogenic diets alter the gut microbiome resulting in decreased intestinal Th17 cells. Cell. (2020) 181:1263–75. doi: 10.1016/j.cell.2020.04.027 PMC7293577 32437658

[17] Sutti S Jindal A Locatelli I Vacchiano M Gigliotti L Bozzola C. Adaptive immune responses triggered by oxidative stress contribute to hepatic inflammation in NASH. Hepatol. (2014) 59:886–97. doi: 10.1002/hep.26749 24115128

[18] Wiede F Tiganis T. Isolation and characterization of mouse intrahepatic lymphocytes by flow cytometry. Methods Mol Biol. (2018) 1725:301–11. doi: 10.1007/978-1-4939-7568-6_23 29322426

[19] Klindworth A Pruesse E Schweer T Peplies J Quast C Horn M. Evaluation of general 16S ribosomal RNA gene PCR primers for classical and next-generation sequencing-based diversity studies. Nucleic Acids Res. (2013) 41:e1. doi: 10.1093/nar/gks808 22933715 PMC3592464

[20] Callahan BJ McMurdie PJ Rosen MJ Han AW Johnson AJ Holmes SP. DADA2: High-resolution sample inference from Illumina amplicon data. Nat Methods. (2016) 13:581–3. doi: 10.1038/nmeth.3869 PMC4927377 27214047

[21] Shearer AM Wang Y Fletcher EK Rana R Michael ES Nguyen N. PAR2 promotes impaired glucose uptake and insulin resistance in NAFLD through GLUT2 and Akt interference. Hepatology. (2022) 76:1778–93. doi: 10.1002/hep.32589 PMC9669194 35603482

[22] Trzaskalski NA Fadzeyeva E Mulvihill EE. Dipeptidyl Peptidase-4 at the interface between inflammation and metabolism. Clin Med Insights Endocrinol Diabetes. (2020) 13:1179551420912972. doi: 10.1177/1179551420912972 32231442 PMC7088130

[23] Vallianou N Christodoulatos GS Karampela I Tsilingiris D Magkos F Stratigou T. Understanding the role of the gut microbiome and microbial metabolites in non-alcoholic fatty liver disease: Current Evidence and Perspectives. Biomolecules. (2021) 12:56. doi: 10.3390/biom12010056 35053205 PMC8774162

[24] Brandl K Schnabl B. Intestinal microbiota and nonalcoholic steatohepatitis. Curr Opin Gastroenterol. (2017) 33:128–33. doi: 10.1097/MOG.0000000000000349 PMC5662009 28257306

[25] Abenavoli L Giubilei L Procopio AC Spagnuolo R Luzza F Boccuto L. Gut microbiota in non-alcoholic fatty liver disease patients with inflammatory bowel diseases: A complex interplay. Nutrients. (2022) 14:5323. doi: 10.3390/nu14245323 36558483 PMC9785319

[26] Zhou Y Zhang F Mao L Feng T Wang K Xu M. Bifico relieves irritable bowel syndrome by regulating gut microbiota dysbiosis and inflammatory cytokines. Eur J Nutr. (2023) 62:139–55. doi: 10.1007/s00394-022-02958-0 PMC9899748 35918555

[27] Silva YP Bernardi A Frozza RL. The role of short-chain fatty acids from gut microbiota in gut-brain communication. Front Endocrinol (Lausanne). (2020) 11:25. doi: 10.3389/fendo.2020.00025 32082260 PMC7005631

[28] Mackinnon AC Tonev D Jacoby B Pinzani M Slack RJ. Galectin-3: therapeutic targeting in liver disease. Expert Opin Ther Targets. (2023) 27:779–91. doi: 10.1080/14728222.2023.2258280 37705214

[29] Peiseler M Schwabe R Hampe J Kubes P Heikenwälder M Tacke F. Immune mechanisms linking metabolic injury to inflammation and fibrosis in fatty liver disease - novel insights into cellular communication circuits. J Hepatol. (2022) 77:1136–60. doi: 10.1016/j.jhep.2022.06.012 35750137

[30] Pugliese N Plaz Torres MC Petta S Valenti L Giannini EG Aghemo A. Is there an ‘ideal’ diet for patients with NAFLD? Eur J Clin Invest. (2022) 52:e13659. doi: 10.1111/eci.13659 34309833

[31] Raza S Rajak S Upadhyay A Tewari A Anthony Sinha R. Current treatment paradigms and emerging therapies for NAFLD/NASH. Front Biosci. (2021) 26:206–37. doi: 10.2741/4892 PMC7116261 33049668

[32] Mooli RGR Ramakrishnan SK. Emerging role of hepatic ketogenesis in fatty liver disease. Front Physiol. (2022) 13:946474. doi: 10.3389/fphys.2022.946474 35860662 PMC9289363

[33] Chatterjee P Fernando M Fernando B Dias CB Shah T Silva R. Potential of coconut oil and medium chain triglycerides in the prevention and treatment of Alzheimer’s disease. Mech Ageing Dev. (2020) 186:111209. doi: 10.1016/j.mad.2020.111209 31953123

[34] Fletcher JA Deja S Satapati S Fu X Burgess SC Browning JD. Impaired ketogenesis and increased acetyl-CoA oxidation promote hyperglycemia in human fatty liver. JCI Insight. (2019) 5:e127737. doi: 10.1172/jci.insight.127737 31012869 PMC6629163

[35] Paoli A Bianco A Moro T Mota JF Coelho-Ravagnani CF. The effects of ketogenic diet on insulin sensitivity and weight loss, which came first: the chicken or the egg? Nutrients. (2023) 15:3120. doi: 10.3390/nu15143120 37513538 PMC10385501

[36] Ahrén B. Glucose-lowering action through targeting islet dysfunction in type 2 diabetes: Focus on dipeptidyl peptidase-4 inhibition. J Diabetes Investig. (2021) 12:1128–35. doi: 10.1111/jdi.13564 PMC8264410 33949781

[37] Carbone LJ Angus PW Yeomans ND. Incretin-based therapies for the treatment of non-alcoholic fatty liver disease: A systematic review and meta-analysis. J Gastroenterol Hepatol. (2016) 31:23–31. doi: 10.1111/jgh.13026 26111358

[38] Hendrikx T Porsch F Kiss MG Rajcic D Papac-Miličević N Hoebinger C. Soluble TREM2 levels reflect the recruitment and expansion of TREM2+ macrophages that localize to fibrotic areas and limit NASH. J Hepatol. (2022) 77:1373–85. doi: 10.1016/j.jhep.2022.06.004 35750138

[39] Asrih M Altirriba J Rohner-Jeanrenaud F Jornayvaz FR. Ketogenic diet impairs FGF21 signaling and promotes differential inflammatory responses in the liver and white adipose tissue. PloS One. (2015) 10:e0126364. doi: 10.1371/journal.pone.0126364 25973847 PMC4431718

[40] Ioannou GN. The role of cholesterol in the pathogenesis of NASH. Trends Endocrinol Metab. (2016) 27:84–95. doi: 10.1016/j.tem.2015.11.008 26703097

[41] Lee G You HJ Bajaj JS Joo SK Yu J Park S. Distinct signatures of gut microbiome and metabolites associated with significant fibrosis in non-obese NAFLD. Nat Commun. (2020) 11:4982. doi: 10.1038/s41467-020-18754-5 33020474 PMC7536225

[42] Tavella T Rampelli S Guidarelli G Bazzocchi A Gasperini C Pujos-Guillot E. Elevated gut microbiome abundance of Christensenellaceae, Porphyromonadaceae and Rikenellaceae is associated with reduced visceral adipose tissue and healthier metabolic profile in Italian elderly. Gut Microbes. (2021) 13:1–19. doi: 10.1080/19490976.2021.1880221 PMC7889099 33557667

[43] Al-Lahham S Roelofsen H Rezaee F Weening D Hoek A Vonk R. Propionic acid affects immune status and metabolism in adipose tissue from overweight subjects. Eur J Clin Invest. (2012) 42:357–64. doi: 10.1111/j.1365-2362.2011.02590.x 21913915

[44] Yang H Meng L Ai D Hou N Li H Shuai X. Acetic acid alleviates the inflammatory response and liver injury in septic mice by increasing the expression of TRIM40. Exp Ther Med. (2019) 17:2789–98. doi: 10.3892/etm.2019.7274 PMC6425238 30906467

[45] Moore MP Cunningham RP Davis RAH Deemer SE Roberts BM Plaisance EP. A dietary ketone ester mitigates histological outcomes of NAFLD and markers of fibrosis in high-fat diet fed mice. Am J Physiol Gastrointest Liver Physiol. (2021) 320:G564–72. doi: 10.1152/ajpgi.00259.2020 PMC8238172 33501889

[46] Liao YJ Wang YH Wu CY Hsu FY Chien CY Lee YC. Ketogenic diet enhances the cholesterol accumulation in liver and augments the severity of CCl4 and TAA-induced liver fibrosis in mice. Int J Mol Sci. (2021) 22:2934. doi: 10.3390/ijms22062934 33805788 PMC7998170

[47] Teratani T Tomita K Suzuki T Oshikawa T Yokoyama H Shimamura K. A high-cholesterol diet exacerbates liver fibrosis in mice via accumulation of free cholesterol in hepatic stellate cells. Gastroenterol. (2012) 142:152–164.e10. doi: 10.1053/j.gastro.2011.09.049 21995947

[48] Kisseleva T Brenner D. Molecular and cellular mechanisms of liver fibrosis and its regression. Nat Rev Gastroenterol Hepatol. (2021) 18:151–66. doi: 10.1038/s41575-020-00372-7 33128017

[49] Puchalska P Martin SE Huang X Lengfeld JE Daniel B Graham MJ. Hepatocyte-macrophage acetoacetate shuttle protects against tissue fibrosis. Cell Metab. (2019) 29:383–398.e387. doi: 10.1016/j.cmet.2018.10.015 30449686 PMC6559243

[50] Vuscan P Kischkel B Joosten LAB Netea MG. Trained immunity: General and emerging concepts. Immunol Rev. (2024) 323:164–85. doi: 10.1111/imr.13326 38551324

[51] Mitroulis I Ruppova K Wang B Chen LS Grzybek M Grinenko T. Modulation of myelopoiesis progenitors is an integral component of trained immunity. Cell. (2018) 172:147–161.e12. doi: 10.1016/j.cell.2017.11.034 29328910 PMC5766828

[52] Li X Wang H Yu X Saha G Kalafati L Ioannidis C. Maladaptive innate immune training of myelopoiesis links inflammatory comorbidities. Cell. (2022) 185:1709–27. doi: 10.1016/j.cell.2022.03.043 PMC9106933 35483374

[53] Crosby L Davis B Joshi S Jardine M Paul J Neola M. Ketogenic diets and chronic disease: weighing the benefits against the risks. Front Nutr. (2021) 8:702802. doi: 10.3389/fnut.2021.702802 34336911 PMC8322232

