# Supplementary material for: Vegetal oil-based ketogenic diet improves inflammation and fibrosis in experimental metabolic dysfunction-associated steatohepatitis
Source: Front Immunol. 2025 Apr 1;16:1518687. doi: 10.3389/fimmu.2025.1518687 (PMC11996634; doi:10.3389/fimmu.2025.1518687)
Supplement: Supplementary file 1 [file DataSheet1.docx]

**Vegetal oil based ketogenic diet improves experimental metabolic dysfunction-associated steatohepatitis (MASH) in mice.**

**Supplementary materials**

**Supplementary methods**

*Metabolomic Analysis.* Plasma SCFAs were extracted with methyl tert-butyl ether (MTBE). Briefly, 50 uL of plasma was placed in a tube, then 2,5 μL of the internal standard propanoic acid D2 and acetic acid D4 (20.4 ppm) were added, and the sample was vortexed for 20 s, followed by spin centrifugation for 15 s. The sample was then brought to pH 2 using 6 M HCl. 140 μL of MTBE was added, and the tube was placed on a rotator for 15 min, followed by centrifugation for 10 min at 4 °C and 21.1×g. Then, 100 μL of the organic phase containing the SCFAs was analyzed with GCxGC-TOFMS analysis. For the analysis, a LECO Pegasus BT 4D GCxGC-TOFMS instrument (Leco Corp., St. Josef, MI, USA) equipped with a LECO dual-stage quad jet thermal modulator was used. The GC part of the instrument was an Agilent 7890 gas chromatograph (Agilent Technologies, Palo Alto, CA), equipped with a split/splitless injector. The first dimension column was a 30 m DB-FATWAX-UI (Agilent Technologies, Santa Clara, CA) with a diameter of 0.25 mm and a film thickness of 0.25 μm, and the second dimension chromatographic columns was a 2 m Rxi-17Sil MS (Restek Corp., Bellefonte, PA) with a diameter of 0.25 mm and a film thickness of 0.25 μm. High-purity helium (99,9999%) was used as the carrier gas with a flow rate of 1.4 mL/min. 1 μL of sample was injected in split less mode at 250°C. The temperature program was as follows: the initial temperature was 40°C for 2 minutes, then ramped 7°C/min up to 165°C, 25°C/min up to 240°C, maintained for 5 minutes. The secondary column was maintained at +5°C relative to the GC oven temperature of the first column. Electron impact ionization was applied (70 eV). The ion source temperature was set at 250°C, the mass range was 40 to 300 m/z with an extraction frequency of 32 kHz. The acquisition rates were 200 spectra/s. The modulation periods were 4s for the entire run. The modulator temperature offset was set at +15°C relative to the secondary oven temperature, while the transfer line was set at 280°C. The chromatograms were acquired in TIC (total ion current) mode. Peaks with signal to- noise (S/N) value lower than 500.0 were rejected. ChromaTOF version 5.31 was used for raw data processing. Mass spectral assignment was performed by matching with NIST MS Search 2.3 libraries adding Fiehn Library. Commercial mix standard of free fatty acids composed by acetic acid, propanoic acid, propanoic acid 2-methyl, butanoic acid, butanoic acid 3-methyl and pentanoic acid was run individually and EI spectra were matched against the NIST library. The calibration curves of the SCFAs were obtained using Excel, while the analytical results were processed and compared with the open-source software MetaboAnalyst 5.0.

*Liver Proteomics Analysis.* For protein extraction 50 mg of liver was homogenized in 500 𝜇L of buffer (7 M urea, 2 M thiourea, 4% (w/v) dimethyl [3 propyl] azaniumyl propane- 1-sulfonate (CHAPS), 1% (v/v) immobilized pH gradient (IPG) buffer pH 3–10 NL), 40 mM dithiothreitol (DTT), and Protease Inhibitor Cocktail Complete) (Roche, Mannheim, Germany) and left at room temperature for 1 hour. Then, the sample was centrifuged at 40,000 ×g for 60 min at 4∘C, the supernatant was removed, and protein concentration was measured using the Bradford protein assay. Fifty micrograms (50 µg) of proteins were subjected to reduction with DTT, alkylation with iodoacetamide and tryptic digestion at 37 ◦C overnight. Peptides were then desalted on the Discovery® DSC-18 solid phase extraction (SPE) 96-well plate (25 mg/well) (Sigma-Aldrich Inc., St. Louis, MO, USA) and then analyzed by label-free LC–MS/MS. Samples were analyzed in two phases: a data-dependent acquisition (DDA) followed by a data-independent analysis (DIA) on the same sample using the same gradient conditions. All samples were analyzed with a micro-LC Eksigent Technologies (Eksigent, Dublin, USA) system interfaced with a 5600+ TripleTOF system (AB Sciex, Concord, Canada) equipped with DuoSpray Ion Source and CDS (Calibrant Delivery System). Peptides were separated using Halo C18 column (0.5×100 mm, 2.7 μm; Eksigent Technologies Dublin, USA). The reverse phase LC solvents include solvent A (99.9% water +0.1% formic acid) and solvent B (99.9% acetonitrile +0.1% formic acid). A 30 min gradient was used at a flow rate of 15 μL/min with an increasing concentration of solvent B from 2% to 40%. For DDA acquisition, experiments were set to obtain a high-resolution TOF-MS scan over a mass range of 100–1500 m/z, followed by an MS/MS product ion scan from 200 to 1250 Da (accumulation time of 5.0 ms) with the abundance threshold set at 30 cps. The ion source parameters in electrospray positive mode were set as follows: curtain gas (N2) at 25 psig, nebulizer gas GAS1 at 25 psig, and GAS2 at 20 psig, ion spray voltage floating (ISVF) at 5000 V, source temperature at 450 °C and declustering potential at 25 V. Using the same conditions as described above, a SWATH acquisition using DIA was carried out for the label-free quantification process. The mass spectrometer was operated so that a 50-ms survey scan (TOF-MS) was performed and subsequent MS/MS experiments were performed on all precursors. These MS/MS experiments were carried out in a cyclical manner using an accumulation time of 40 ms per 25-Da swath (36 swaths in total) for a total cycle time of 1.5408 s. The ions were fragmented for each MS/MS experiment in the collision cell using the rolling collision energy. The MS data were acquired with Analyst TF 1.7 (AB SCIEX, Concord, Canada). Peptides (and proteins) were identified using DDA followed by database search, while the quantification was obtained by integrating the area under the chromatographic peak for each ion fragment of identified peptides by using the DIA file. DDA files were searched against the mouse UniProt Swiss-Prot reviewed database containing mouse proteins (version 20july2020, containing 23354 sequence entries) using Protein Pilot software v. 4.2 (SCIEX, Concord, Canada) and Mascot v. 2.4 (Matrix Science Inc., Boston, USA). Samples were input in the Protein Pilot software with the following parameters: cysteine alkylation, digestion by trypsin, no special factors and False Discovery Rate (FDR) at 1%. For Mascot search, we selected Trypsin as digestion enzyme with 2 missed cleavages, set the instrument to ESI-QUAD-TOF and specified the following modifications for the assay: carbamidomethyl cysteine as fixed modification and oxidized methionine as variable modification. An assay tolerance of 50 ppm was specified for peptide mass tolerance, and 0.1 Da for MS/MS tolerance. The charges of the peptides to search for were set to 2+, 3+, and 4+, and the search was set to monoisotopic. A target-decoy database search was performed, and FDR was fixed at 1%. SwathXtend was employed to build an integrated assay library with the DDA acquisitions to use as the ion library file for all SWATH analysis and quantification. Protein quantification was performed by integrating the extracted ion chromatogram of all the unique ions for a given peptide. Spectral alignment of the SWATH samples (DIA run) was carried out with PeakView 2.2 (ABSCIEX, Concord, Canada) using the spectral library generated above and the following parameters: 6 peptides per protein, 6 transitions per peptide, XIC extraction window of 5 min and a width of 15 ppm. Shared peptides were excluded as well as peptides with modifications. Peptides with FDR lower than 1.0% were exported in MarkerView 1.2 (ABSCIEX, Concord, Canada) for the t-test.

*Real-time PCR* *analysis*. The following TaqMan Gene Expression probes for mouse were used for the analysis: TNF-α (Mm99999068_m1), IL-12p40 (Mm99999067_m1), CD11b (Mm00434455_m1), CCL2 (Mm00441242_m1), TREM-2 (Mm04209422_m1), galectin-3 (Mm00802901_m1), osteopontin (Mm01204014_m1), α1-procollagen (Mm00801666_g1), TGF-β1 (Mm00441724_m1), and beta-actin (Cat. N4352663).

**Supplementary Table 1**

Changes in the liver proteome profiles of mice with MASH induced by feeding Western diet (WD) and switched to Ketogenic diet (KD) as compared to animals remaining on WD.

| **Functions Annotation** | **p-value** | **Activation z-score** | **N° proteins** | **Proteins** |
| --- | --- | --- | --- | --- |
|  |  |  |  |  |
| Lipid Metabolism | 3,47E-23 | 2,136 | 77 | ABCB11,ABCD3,ACAD9,ACADL,ACADM,ACADSB,ACADVL,ACAT1,ACLY,Acot1,ACOT12,ACOT2,ACSL1,ACSL5,ACSM1,ACSM3,AKR1B1,ANXA1,APOB,Apoc3,APOC4,ASAH1,ATP11C,ATP5PF,CANX,CBR1,CD36,CES1,COTL1,CPT1A,CPT2,CRAT,CROT,CYP1A2,CYP27A1,Cyp2c54,CYP2E1,Cyp2j5,Cyp4a14,CYP4A22,ECI2,EGFR,ELAVL1,EPHX1,FAAH,FABP5,FASN,GPX4,GSTM1,HACL1,HMGCS2,IGHM,KNG1,LIPA,LYPLA2,ME1,MGLL,MIF,NAXE,PCCA,PITPNB,PLG,PON1,PPT1,RHOA,SAR1B,SCD,SLC10A1,SLC25A10,SLC25A13,SLC27A2,SLC27A5,SLC9A3R1,SLCO1B3,SPHK2,TECR,THRSP |
| Glucose metabolism | 1,14E-10 | -2,250 | 114 | AADAC,ABCD3,ACADL,ACADVL,ACSL1,ACSM3,ACSS3,ADK,AKR1B1,ALDOB,ANPEP,ANXA1,ANXA5,APOB,Apoc3,ATP1A1,BBOX1,BHMT,CA3,CANX,CD36,CDH2,Ces1g,CISD1,CKB,COL14A1,COL1A1,COL1A2,COL6A1,COL6A2,CPT1A,CROT,CTSC,CTSH,CYP2E1,Cyp4a14,DHFR,DPP4,DPP7,EGFR,ELAVL1,ERAP1,ETFDH,FAAH,FASN,FKBP1A,FKBP4,FMO5,GAA,GANAB,GBE1,GCKR,GLS2,GLUL,GPD2,GRN,GSTA5,H6PD,HACL1,HMGCS2,HP,HSD11B1,HSD17B11,HSPA5,HYOU1,IDO2,IGHM,IMMT,KMO,LCP1,LGALS3,LGALS9B,LGMN,LIPA,MAOB,ME1,MGLL,MGST1,MIF,MTCO2,Mup1,OPA1,PDXK,PGD,PHB,PKLR,PM20D1,PON1,PREP,PSMB5,PSMB8,PSMD12,PSMD7,QPRT,RPL39,RPS15A,RPS24,RPS27L,RPS6,SCD,SERPINA1,SERPINA3,Slc25a1,SLC25A13,SLC25A20,SLC27A2,SLC2A2,SPHK2,STAT1,SUB1,TGFBI,TGM2,UFC1,UGT1A6 |
| Insulin resistance | 2,81E-07 | -2,622 | 32 | ACADL,ACSL1,ADK,Apoc3,ATP1A1,CA3,CD36,Ces1g,DPP4,DPP7,FAAH,FASN,GAA,GBE1,GCKR,GRN,HSD11B1,HYOU1,LIPA,MGLL,MIF,Mup1,QPRT,RPL39,RPS15A,RPS24,RPS27L,SCD,SLC2A2,SPHK2,SUB1,TGM2 |
| Hepatic steatosis | 9,64E-13 | -2,800 | 44 | ABCB11,ACADL,ACADM,ACADVL,ACLY,ACOX2,ACSL1,ADK,AMT,APOB,BHMT,CD36,Ces1g,CNPY2,CPT1A,CPT2,CYP2E1,Cyp4a14,DDC,DPP4,DPP7,EGFR,EIF4A3,FABP5,FASN,FDXR,GFER,GPD2,HINT2,HSD11B1,LGALS3,LIPA,MACROH2A1,ME1,MGLL,PCCA,PDK2,PEX11A,SCD,SERPINA1,SLC25A13,SLC27A5,SLC2A2,SRSF2 |
| Inflammatory response | 3,01E-06 | -1,950 | 70 | ABCB11,ADK,ANXA1,ANXA3,ANXA5,APOB,ATP11C,BSG,CD36,CHCHD2,CKB,COL1A1,COL1A2,COTL1,CROCC,CYB5B,CYP2E1,DCN,DHFR,DPP4,EGFR,EIF4A3,FAAH,FASN,FDXR,FKBP1A,GAA,GAPDH,GNAI2,GRN,GSDMD,GSTM1,HP,HPX,HSD11B1,HSPA5,IGHM,KMO,LGALS1,LGALS3,LGALS9B,LIPA,MGLL,MIF,MPC1,MT-CO2, Mug1/Mug2,PEX11A,PHB,PLG,PON1,PON3,PPP2CA,PSMB5,PSMB8,PSMD1,SAR1B,SCD,SEC61A1,SERPINA1,SERPINA3,SLC10A1,SLC2A2,SRSF2,STAT1,TGM2,TPMT,TUBA4A,TUBB2A,UGT1A1 |

MASH was induced in wildtype C57BL/6 mice by 16 weeks feeding with Western diet (WD) before the switching to ketogenic diet (KD) for further 8 weeks. Reference mice received WD for 24 weeks.

**Supplementary figures**

**
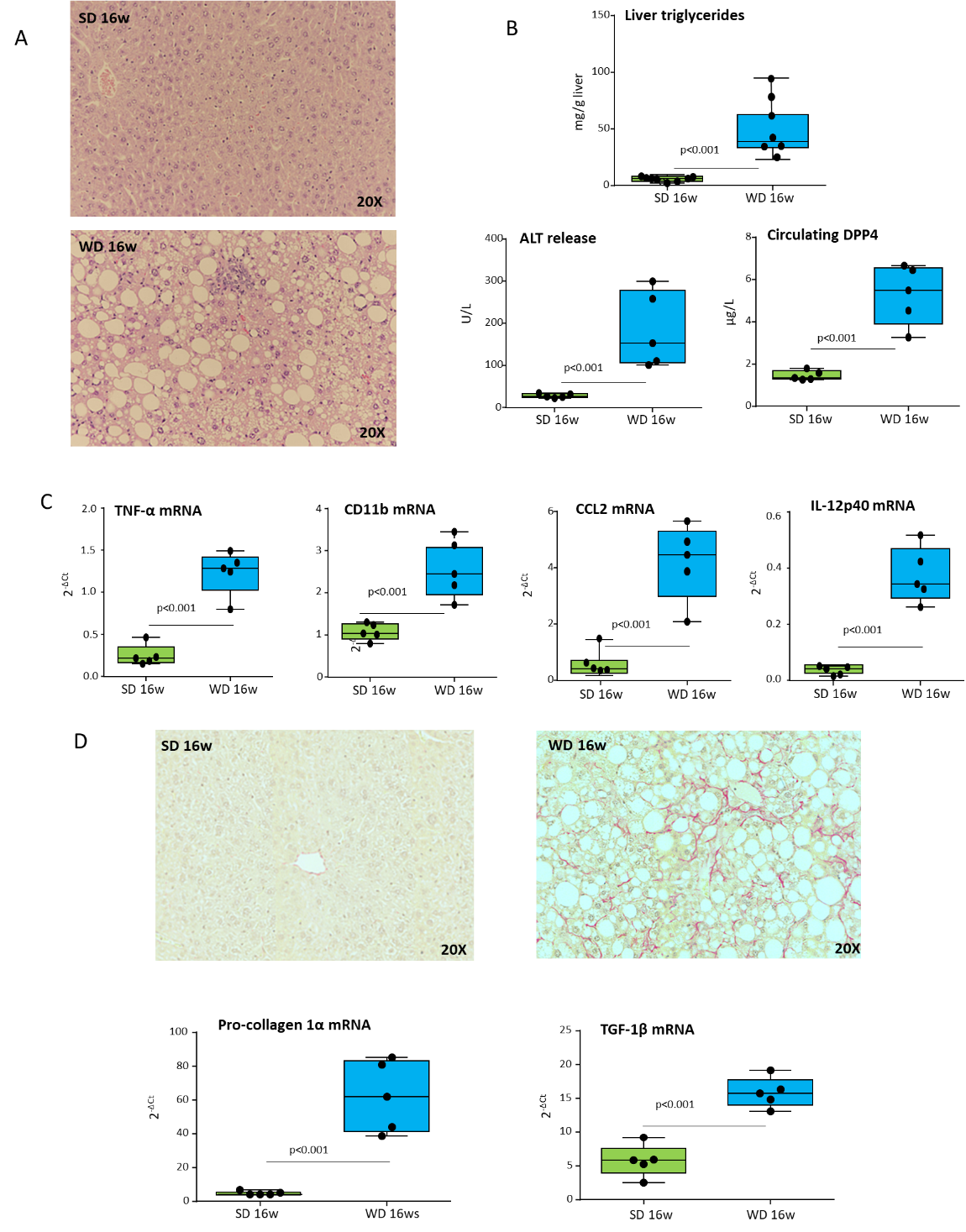
**

**Supplementary Figure 1.** Morphological and biochemical features of MASH induced by mice feeding with a Western diet (WT) for 16 weeks.

(Panel A) Hematoxylin/eosin-staining of liver sections (magnification 20×); (Panel B) Changes in liver triglyceride content and circulating levels of alanine aminotransferase (ALT) and Dipeptidyl Peptidase-4 (DPP4). (Panel C) Hepatic gene expression of pro-inflammatory markers TNF-α, CD11b, CCL2, and IL-12p40. (Panel D) Intrahepatic fibrosis deposition as evidenced by liver sections collagen staining with Sirius Red (magnification 20×) or the hepatic transcripts for TGF-β1 and procollagen-1α. RT-PCR values are expressed as fold increase of 2^-ΔCT^ after normalization to the β-actin gene. The boxes include the values within the 25th and 75th percentile, while the horizontal bars represent the medians. The extremities of the vertical bars (10th–90th percentile) comprise 80% of the values.

**
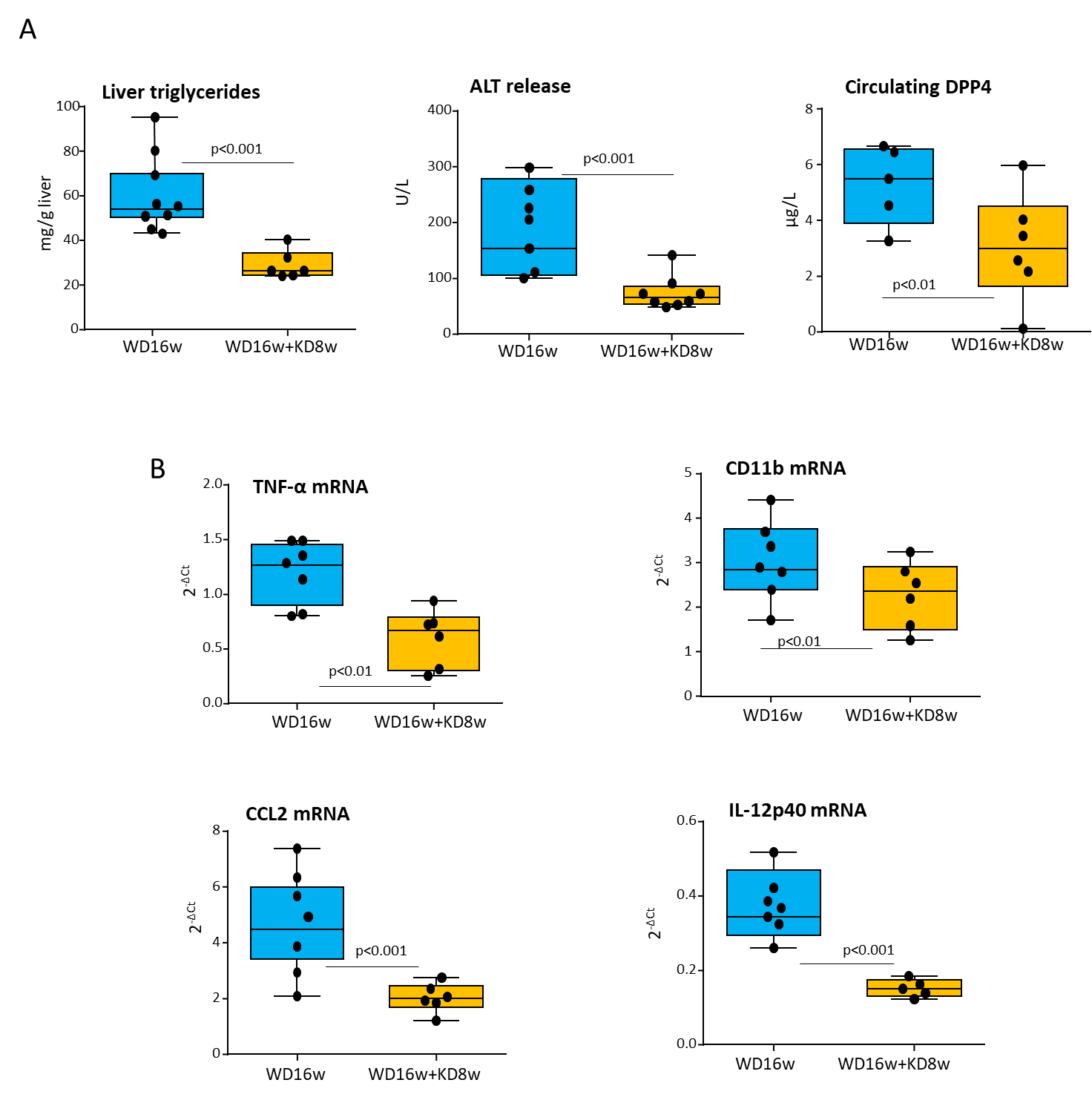
**

**Supplementary Figure 2**. Changes steatosis and hepatic injury following mice switching to ketogenic diet.

MASH was induced in wildtype C57BL/6 mice by 16 weeks feeding with Western diet before the switching to ketogenic diet for further 8 weeks (WD16w+KD8w). Reference mice received WD for 16 weeks (WD16w). (Panel A) Changes in liver triglyceride content, circulating levels of alanine aminotransferase (ALT) and circulating Dipeptidyl Peptidase-4 (DPP4). (Panel B) Hepatic gene expression of pro-inflammatory markers TNF-α, CD11b, CCL2, and IL-12p40. RT-PCR values are expressed as fold increase of 2^-ΔCT^ after normalization to the β-actin gene. The boxes include the values within the 25th and 75th percentile, while the horizontal bars represent the medians. The extremities of the vertical bars (10th–90th percentile) comprise 80% of the values.

**
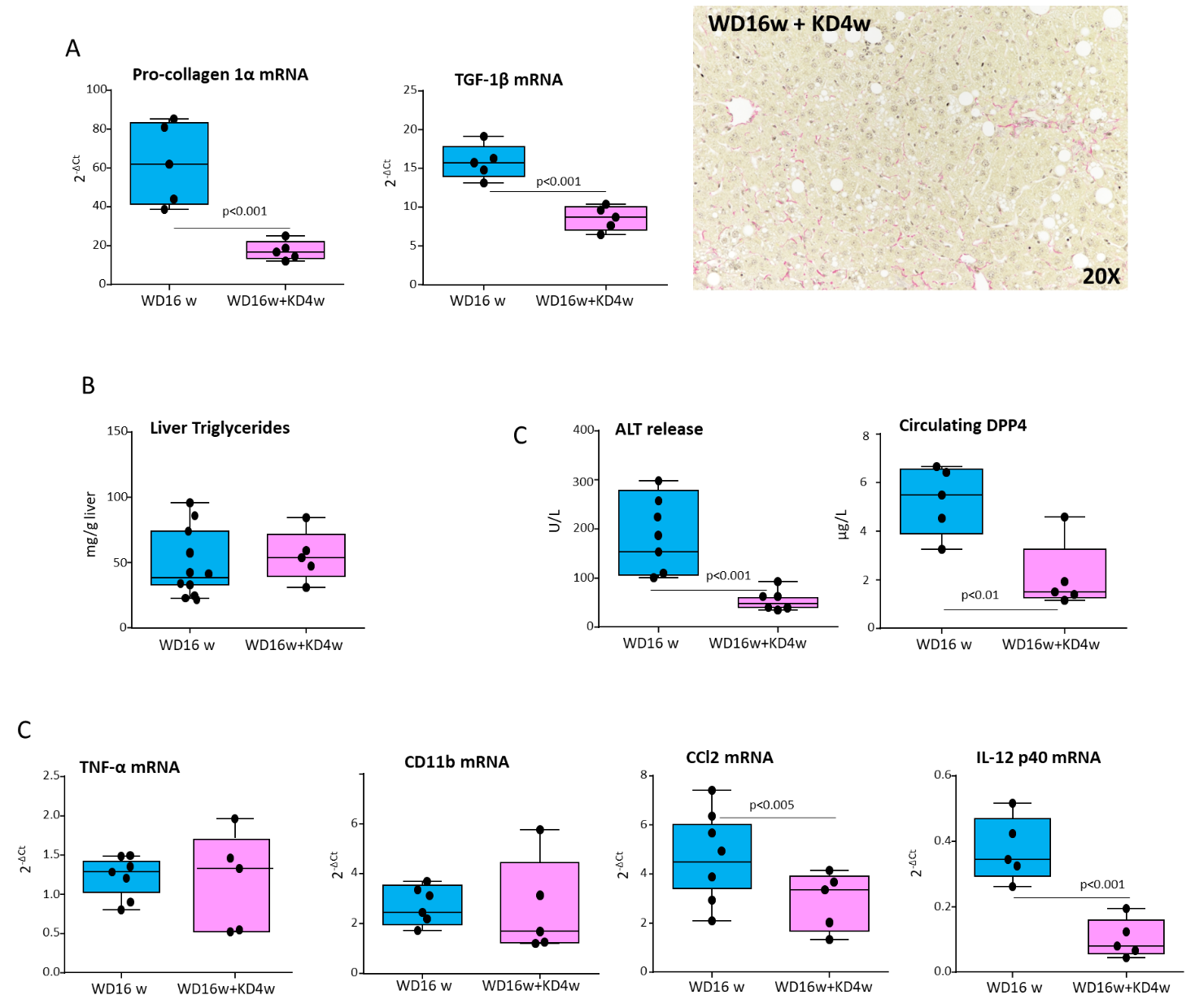
**

**Supplementary Figure 3**. Short-term switching on ketogenic improves MASH-associated liver injury and fibrosis.

MASH was induced in wildtype C57BL/6 mice by 16 weeks feeding with Western diet before the switching to ketogenic diet for further 4 weeks (WD16w+KD4w). Reference mice received WD for 16 weeks (WD16w). (Panel A) Hepatic transcripts for pro-fibrogenic markers TGF-β1 and procollagen-1α and intrahepatic collagen staining with Sirius Red (magnification 20×). (Panel B) Changes in liver triglyceride content, circulating levels of alanine aminotransferase (ALT) and circulating Dipeptidyl Peptidase-4 (DPP4). (Panel C) Hepatic gene expression of pro-inflammatory markers TNF-α, CD11b, CCL2, and IL-12p40. RT-PCR values are expressed as fold increase of 2^-ΔCT^ after normalization to the β-actin gene. The boxes include the values between the 25th and 75th percentile, while the horizontal bars represent the medians. The extremities of the vertical bars (10th–90th percentile) comprise 80% of the values.
